# Pollen transfer and patterns of reproductive success in pure and mixed populations of nectariferous *Platanthera bifolia* and *P. chlorantha* (Orchidaceae)

**DOI:** 10.7717/peerj.13362

**Published:** 2022-06-13

**Authors:** Emilia Brzosko, Krzysztof Frąckiel, Edyta Jermakowicz, Paweł Mirski, Beata Ostrowiecka, Izabela Tałałaj

**Affiliations:** 1 Department of Biology and Plant Ecology, Faculty of Biology, University of Białystok, Białystok, Podlasie, Poland; 2 Biebrza National Park, Osowiec-Twierdza, Goniądz, Podlasie, Poland

**Keywords:** Female reproductive success, Putative hybrids, Moths, Pollen flow, Seed viability, Seed germination, Floral display, Flower traits, Male reproductive success

## Abstract

Plant species evolution is driven by many factors that have different roles in space and time. Using different field and laboratory methods, we studied reproductive patterns and their determinants in pure and mixed *P. bifolia* and *P. chlorantha* populations in different habitats. We also considered the probability of hybridisation between these two species and the role of intra-population processes in maintaining species integrity. Generally, we found a high level of reproductive success in both Platantherans. In both species, male (MRS) and female (FRS) reproductive success depended on floral display, and male reproductive success additionally on population structure. The flower traits were only weakly related to reproductive success. Moths’ assemblages varied spatially and temporally, and their diversity and numbers were correlated with MRS in the year, when their abundance was markedly lower. Analysis of patterns of pollen transfer showed that pollen was transported up to 25 m (average 8.2 ± 4.83 m) and showed gene exchange between these two *Platanthera* species. The germination level of both species was significantly lower than seed viability, although *P. bifolia* seed germinated with higher frequency than *P. chlorantha* seeds. We noted differences in viability and germination of seeds developed as an effect of experimental interspecies crossings and those developed from natural pollination. The presence of intermediate ecotypes together with observations of spontaneous interspecies crosses in the field and viability of seeds produced in interspecies crossing suggest that both pre- and postzygotic reproductive barriers are not complete and do not prevent hybrid production.

## Introduction

Orchidaceae exhibits the full continuum of pollination systems from highly specialised to generalised, and the effectiveness of pollination of orchids depends on specialisation level (Joffard et al., 2019; Philips, Reiter & Peakall, 2020). Orchids are known as one of the most specialised plants with respect to their pollinators—the majority of them (67%) are pollinated by single pollinator species (Tremblay, 1992) or one functional group of pollinators. Such rigorous specialisation requires a mutual match between flower and pollinator morphologies for effective pollination (Moré et al., 2012). Therefore, flower traits play a crucial role in attracting pollinators and shaping reproductive success, which is documented in many studies, including those on long-spurred plants (Inoue, 1986; Nilsson, 1988; Maad, 2000; Alexandersson & Johnson, 2002; Little, Dieringer & Romano, 2005; Whittall & Hodges, 2007; Boberg & Ågren, 2009; Boberg et al., 2014; Trunschke, Sletvold & Ågren, 2018). The example of long-spurred orchids are the objects of our studies—*Platanthera bifolia* and *P. chlorantha*, the most common representatives of *Platanthera* genus in Europe, occurring in sympatry. Their flowers differ in column structure, floral scent (Nilsson, 1983, 1985; Tollsten & Bergström, 1993; Esposito et al., 2018) and nectar chemistry (Brzosko & Bajguz, 2019). According to the results of recent studies, the most important flower trait in attracting pollinators, thus in determining the fitness of these orchids, is spur length (Nilsson, 1983; Maad & Nilsson, 2004; Boberg et al., 2014).

Flower properties differ between species and distinct populations of one species (Maad & Nilsson, 2004; Bateman & Sexton, 2008; Boberg et al., 2014; Brzosko & Bajguz, 2019). These differences might be because plants growing in contrasting habitats may show local adaptation of flowers to biotic and abiotic conditions (Boberg et al., 2014; Jacquemyn et al., 2017), which may be reflected in reproductive success (RS) (Johnson & Bond, 1992; Huda & Wilcock, 2008; Moré et al., 2012; Boberg et al., 2014). Another important trait in shaping RS is the floral display. Plants tend to evolve more attractive flowers/inflorescences to increase the attraction for pollinators. In nectariferous species, larger inflorescences offer more rewards due to the aggregation of resources (*e.g*. nectar) and influence pollinators’ behaviour. They stay longer on a single inflorescence, visit more flowers and reduce travel time (and save energy) between food sources. Thus, a larger floral display increases the individual fitness (Ohashi & Yahara, 1999; Brzosko, 2002, 2003; Grindeland, Sletvold & Ims, 2005; Vallius, Arminen & Salonen, 2006; Huda & Wilcock, 2008), but may cause inbreeding depression (Sletvold et al., 2012; Tao et al., 2018). Floral display may be also considered at the population level. RS is often related to population size and in small populations (common in orchids) is often low due to the pollen-limitation (Huda & Wilcock, 2008; Pérez-Hérnandez et al., 2011; Gijbels et al., 2015; Brzosko et al., 2017). Also, spatial distribution of plants shapes the quantity and quality of progeny by influencing pollinators’ behaviour (Internicola et al., 2006), which affect plant mating patterns (Grindeland, Sletvold & Ims, 2005; Nattero et al., 2011). Pollinators are better attracted by denser patches, where the probability that relatives exist is higher, increasing the frequency of crosses between them or selfing, especially in rewarding species. On the whole, this may negatively influence the quantity and quality of the progeny.

The differences in column structure are connected with distinct pollinaria attachment—tongue-attachment in *P. bifolia* and eye-attachment in *P. chlorantha* (Nilsson, 1983; Maad & Nilsson, 2004). In effect, different species have been found as main pollinators of *P. bifolia* and *P. chlorantha*, and their assemblages also depend on geography and vegetation types (Nilsson, 1983; Bateman & Sexton, 2008; Boberg et al., 2014; Esposito, Merckx & Tyteca, 2017; Mõtlep et al., 2021). Despite the fact that flower traits constitute reproductive isolation, both Platantherans, at least partially, share pollinators, which increases the probability of hybridisation. Their hybrids have been found in different parts of geographic ranges (Nilsson, 1983, 1985; Bateman & Sexton, 2008; Schiestl & Schlüter, 2009; Moré et al., 2012; Esposito, Merckx & Tyteca, 2017; Esposito et al., 2018; Mõtlep et al., 2021). Hybridisation is a common phenomenon in plants and is often observed in orchids, which highlights its importance in evolutionary processes (Campbell & Aldridge, 2006; Schiestl & Schlüter, 2009; Xu et al., 2011; Baack et al., 2015; Esposito et al., 2018; Mõtlep et al., 2021).

Although knowledge about the effect of pollinator- and habitat-mediated selection on flower traits (in result on RS) and their role in plant species evolution has increased in recent years, full understanding of these problems require further study. Areas where sister species grow together and share pollinators are especially valuable in this context because they provide a special “laboratory”, in which the process of species divergence and speciation may be explained (Hewitt, 1988). Thus, studies on closely related species that occur in sympatry are important to understanding how species boundaries are maintained or eroded (Wallace, 2006). An example of a suitable evolutionary model system, offering a unique opportunity to study the above-mentioned problems are *P. bifolia* and *P. chlorantha*. They have been the objects of studies on species biology (especially pollination biology) and demography (Nilsson, 1983, 1985; Maad, 2000; Brzosko, 2003; Maad & Alexandersson, 2004; Maad & Nilsson, 2004; Maad & Reinhammar, 2004; Bateman & Sexton, 2008; Claessens & Kleynen, 2011; Bateman, James & Rudall, 2012; Steen, 2012; Boberg et al., 2014; Sexton, 2014; Mõtlep et al., 2018), genetic diversity (Brzosko et al., 2009; Brzosko & Wróblewska, 2013) and hybridisation (Durka et al., 2017; Esposito, Merckx & Tyteca, 2017; Esposito et al., 2018; Mõtlep et al., 2021). Because populations from NE Poland are located more centrally in the geographical range (under distinct climate and habitat conditions), plant-pollinator interactions, and in effect reproduction may be modified. Studies conducted in different habitats and populations within a species range enrich knowledge on this topic because we do not know where and when evolutionary processes operate with sufficient pressure to achieve an important step in species evolution. Moreover, studies at the population level are crucial because all changes at the species level are a consequence of accumulated changes within populations.

Taking into account the above-mentioned problems, we applied a wide set of methods to determine: (1) differences in reproductive patterns and their determinants between the two sister species, (2) the role of pollinators in *P. bifolia* and *P. chlorantha* populations, their assemblages and efficiency as pollen vectors, (3) the probability of hybridisation between two species and the role of reproductive processes in maintaining species integrity.

## Materials and Methods

### Study species

*P. bifolia* (L.) L. C. Rich. and *P*. *chlorantha* (Cust.) Rchb. are closely related and widely distributed species, which largely occur in sympatry (Hultén & Fries, 1986). In Poland, *P. bifolia* has a wider distribution than *P. chlorantha*. The studied species inhabit a wide range of habitats (Claessens & Kleynen, 2011). They overlap in flowering. The inflorescences produce 10–20 white flowers with spurs, in which nectar is secreted. *P. bifolia* has a narrow column, and pollinaria are parallel and close to each other, while the *P. chlorantha* column is wider, and the pollinaria are further apart and positioned at an angle (Nilsson, 1983, 1985; Claessens & Kleynen, 2011; Boberg et al., 2014). The caudicle in *P. bifolia* is much shorter than in *P. chlorantha* and sufficient to avoid self-pollination (Nilsson, 1983). The species also differ in terms of floral scent (Nilsson, 1983; Tollsten & Bergström, 1993; Esposito et al., 2018) and nectar chemistry (Brzosko & Bajguz, 2019). They are adapted to pollination through Lepidoptera, predominantly by sphingid and noctuid moths (Nilsson, 1983; Claessens & Kleynen, 2011). Platantherans can hybridise, and pollen transfer always takes place from a flower with a shorter spur (*P. bifolia*) to a flower with a longer spur (*P. chlorantha*) (Nilsson, 1985). Both species are predominantly allogamous (Nilsson, 1978, 1983; Tałałaj et al., 2017), but spontaneous autogamy was also noted (Brzosko, 2003).

### Study area and populations

Our studies were performed in three regions of NE Poland and included populations from Białowieża National Park (BNP; two populations: BC and BF), Biebrza National Park (BbNP; one population: POG), and Suwałki Landscape Park (SLP; four populations: SMOL, LIN, BON, and POB) (all field research and laboratory experiments were approved by appropriate institution: The Environmental Ministry of Poland (DOP-WPN.286.53.2017MŚ), The Regional Director of Environmental Protection (WPN.6400.40.2016.MW, WPN.6400.15.2017.MR) and Biebrza National Park (*e.g*. 59a/2017)). These three areas are at a distance of 150–200 km, and have different characteristics (*e.g*. in climatic conditions and habitats). SLP, the most northern region, is characterised by the most severe weather conditions (lowest temperatures and shortest vegetation season). Populations existed in different habitats—from open (*e.g*. two meadow populations, LIN and BC, exposed more than the others to the wind and sun) to forests (Table 1). Overall, three pure *P. chlorantha*, two pure *P. bifolia*, and two mixed populations were investigated. In NE Poland, *P. bifolia* is more frequent, but in BNP, *P. chlorantha* dominates. In BbNP, only *P. bifolia* is present. In SLP, both species exist, and mixed populations were observed (POB1+POB2 and SMOLF). In the mixed populations POB1+POB2, *P. chlorantha* individuals (POB2) were less frequent and located at the border of the POB1 *P. bifolia* population. In the SMOLF *P. bifolia* population, one *P. chlorantha* individual was found.

**Table 1:** Habitats characteristics of *Platanthera bifolia* and *P. chlorantha* studied populations.

REGION	Population	Size (ha)	Habitat	Plant community type	Visibility of Platantherans	Understory cover (%)
SLP (Suwałki LP)	SMOLF	~150	Forest	Fragment of birch-spruce forest surrounded by multispecies meadows in agriculture landscape	Higher than other plants	10–40
	SMOLM	~200	Open	Rich multispecies meadow, grazing	Equal	100
	LIN	~150	Open	Rich multispecies meadow, grazing	Equal	100
	BON	~60	Forest	Hornbeam-oak forest	Higher than other plants	20–70
	POB1	~50	Artificial young forest copse	Artificial dense young spruce community without plants under spruces and with small gaps among tree aggregations dominated by grasses	Higher than other plants	0–100
	POB2	~2-	Artificial young forest copse	Dense young spruce plantation without plants under spruces and with small gaps among tree aggregations dominated by grasses	Higher than other plants	80
BbPN (Biebrza NP)	POG	~50	Open/Forest	Border of peat bogs with single trees and shrubs	Higher than other plants/ equal	80–100
BPN (Białowieża NP)	BF	~70	Forest	Hornbeam-oak forest	Higher than other plants	10–50
	BC	~200	Open	Rich multispecies meadow among forests	Equal	100

All populations from the SLP were studied over 3 years (2015–2017), and in 2016, one *P. chlorantha* population from the BNP and one *P. bifolia* population from the BbNP were included to analyse flower traits. In 2017, seven populations of both species from three regions were fully investigated. Each year, all individuals from the populations were included, except for the largest SMOL *P. bifolia* population, which was located at the edge of the meadow and forest, partially under the tree canopy. Over 3 years, we investigated populations in forested areas, and in 2017, only 30 individuals were included from the meadow to check whether the plants’ RS depends on the habitat type (open SMOLM *vs*. forest SMOLF).

### Levels and determinants of reproductive success

#### Density and spatial patterns of individuals in P. bifolia and P. chlorantha populations

All individuals in the populations were mapped in the field using scaled tape and millimetre paper. If the distance between the closest individuals was greater than 30 m, a handheld GPS was used to map with a 60-s averaged location measurement to increase the accuracy. Paper maps were digitised using ArcGIS 10.3 (Esri) and georeferenced using local geographic projection (CS PL 1992). To test the influence of the spatial population structure on RS, we performed the spatial analyses described below. Relative density was calculated using the kernel function in a search radius of 10 m and an output pixel size of 1 m. A distance matrix was created to calculate the mean distance between each shoot and all shoots in the population, and to measure the distance to the nearest shoot. The resulting variables were the shoot kernel density and the mean and minimum distance to neighbouring shoots, which were acquired and later used in models of RS.

#### Floral display and reproductive success

We measured the floral display traits, which may affect pollinator attraction and RS: height of flowering plants, length of inflorescences and number of flowers in the inflorescence. When capsules started to rip, female (FRS, number of fruits) and male (MRS, pollinaria removal) RS were determined and used to calculate the MRS:FRS ratios for each population. Here, the MRS:FRS ratio is considered as intermediate information about the efficiency of insect visitors. To check the influence of flower position on pollination success, we compared the fruit set in the inflorescence, dividing it into the lower and upper parts, and analysed this data using the Mann–Whitney U test.

### Potential reproductive barriers of two sympatric *Platanthera* species

#### Flower traits in the context of putative hybrids and reproductive success

To examine inter- and intra-species variation in flower traits and their influence on RS, one flower from each individual was collected—in six populations in 2016 and in seven in 2017. To avoid variation along the inflorescence, the lowest flower was collected (rarely, if the first flower was damaged, a second flower was collected). Flowers were stored in 75% ethanol. The following flower traits were measured using an opto-digital DSX110 microscope (Olympus, Tokyo, Japan): lip length and width, spur length, spur entrance width, distance between viscidia, and pollinarium length. In 2015 and 2017, the nectar standing crop (nectar column height) was measured in the field using electronic callipers to the nearest 0.01 mm. Flower traits were also used to detect potential hybrids. Mixed model ANOVAs with population as the random factor were used to test the differences in floral display and flower traits between *P. bifolia* and *P. chlorantha*.

To select a set of traits that allowed for the best *a priori* separation between defined groups of *Platanthera* species and putative hybrids, we employed principal components analysis (PCA) on a correlation matrix using flowers morphology data. Analyses were performed using data from 2016 and 2017 for six and seven populations, respectively. The following traits were included in the analyses: lip length and width, spur entrance width, distance between viscidia, and pollinarium length. Traits of potential hybrids (revealed using PCA) were compared with the corresponding population-mean values using a Wilcoxon test.

Factors affecting RS were investigated with generalised linear models (with beta distribution) using the R 3.2.4 software and the *betareg* package (Cribari-Neto & Zeileis, 2010). Chosen predictors were the variables of floral display (shoot height–the distance from the ground to the inflorescence top, inflorescence height, and number of flowers) and spatial population structure (kernel density of shoots and mean and minimum distance to neighbouring shoots). Separate models (due to the smaller sample size of flowers measured in detail) were built to test how the flower traits affected RS. Prior to modelling, variables were scaled using *dplyr* package (Wickham et al., 2018). All models were built using three options, including the following sets of variables: (1) species only; (2) species additive to other variables; and (3) species interacted with other variables. The most supported model was chosen according to the Bayesian Information Criterion (BIC).

#### Pollen flow within and between P. bifolia and P. chlorantha populations

Pollen flow was monitored in SMOLF, LIN, BON and POB1 populations. To follow the pollen flow, four food pigment colours were used (red, green, blue, and orange). Each colour was applied to clusters of neighbouring shoots (growing in a radius of 1 m; see Supplemental Information). On 16 June 2016, 11 and nine shoots from *P. chlorantha* LIN and *P. bifolia* SMOLF populations were included in the experiment, while all inflorescences from POB1 and BON populations were included. An aqueous solution of pigments was carefully applied to the anther cup using a syringe with a thin blood needle until the pollinium absorbed the pigment. Some shoots included in the experiment were damaged; thus, the final number of shoots was lower. In the POB1 and BON populations, only one colour was used (green in POB1 and red in BON). One week later, we checked the stigmas in all flowers in the populations to assess the frequency and distance to which the marked pollen was exported. When on the stigmas of a given inflorescence the same massulae colour was found as that applied onto the pollinia in this inflorescence, we assumed that it was due to pollinator-mediated autogamous or geitonogamous pollination during one visit. The pollen flow distance that we registered should be treated as the minimal value because we assumed that the colour-marked pollen was brought from the nearest shoot using the marked pollinia.

#### Assemblages of moths in P. bifolia and P. chlorantha populations

During the peak of flowering, moths were collected using stationary light traps (in 2016 in all populations from SLP and in 2017 in populations from three regions). Moths in populations from a given region were captured on the same night from 8:00 pm until 8:00 am the next day. The captured moths were checked for the presence of pollinaria. To assess the possibility of pollination in particular populations, we compared the distance from the spur mouth to the upper level of the nectar column with the proboscis length of the captured moths. Due to the small number of undamaged individuals, data on proboscis length mainly relied upon data from Willmer (2011) and Boberg et al. (2014). Since the last authors found no or marginally significant differences in the proboscis length among populations, we hypothesised that these proboscis sizes were similar to those in other regions. Comparison of our measurements with literature data supports this assumption. Differences in moth assemblages were tested using variables (the number of individuals, species diversity of all moths caught and potential pollinators according to literature data) between the open and forested biotopes using the Wilcoxon test and between populations using the Kruskal–Wallis test.

### Compatibility experiment, seed viability, and germination

An interspecies hand pollination experiment was conducted each year to test the compatibility between two *Platanthera* species. In 2015 and 2016, six *P. bifolia* inflorescences were experimentally pollinated using *P. chlorantha* pollen and nine *P. chlorantha* inflorescences were pollinated using *P. bifolia* pollen. To test the viability of seeds produced in different populations and to compare viability of seeds between the natural fruit set and fruit set from interspecies hand pollination groups, we collected the ripe fruits. We collected all fruits from hand-pollination experiments, and for natural pollination one or two capsules per shoot that were taken that were located at the lowest position on the inflorescence. Over 2 years we collected 85 fruits from *P. bifolia* natural pollination and 134 fruits from *P. chlorantha* natural pollination. From interspecific experiments, 44 fruits were collected and underwent the *in vitro* germination procedure. Capsules were stored at room temperature for 12 weeks and then stratified at 4 °C for 16 weeks. Before sowing, seeds were sterilised for 20 min in 5% Ca(OCl)_2_ with a drop of Tween 80 (detergent) and rinsed six times in sterile distilled water. We then used a modified Malmgren’s medium for terrestrial orchids (Malmgren, 1996; Pierce et al., 2010) that was prepared according to the following protocol: agar 6 g·L^−1^ (Biocorp, France), sucrose 10 g·L^−1^ (Poch, Flint, MI, USA), activated charcoal 0.5 g·L^−1^ (Sigma, St. Louis, MO, USA), (Ca)_3_PO_4_ 75 mg·L^−1^, KH_2_PO_4_ 75 mg·L^−1^, MgSO_4_ · 7 H_2_O 75 mg·L^−1^, NH_4_NO_3_ 100 mg L^−1^, NH_4_H_2_PO_4_ 150 mg L^−1^ (Chempur, Karlsruhe, Germany), coconut milk 50 ml·L^−1^ (Tao Tao), and pH 5.8. Sterile media were dispensed into 50-mm diameter Petri dishes. Seeds from each capsule were inoculated onto three Petri dishes (subsamples), with approximately 300 seeds per subsample. After the Petri dishes were sealed using a double layer of Nescofilum (Bemis, USA), they were cultivated at 21 ± 2 °C in the dark for 40 days. The growth dishes were tested after incubation for progeny quality as follows: (1) seed viability including (a) the number of aborted seeds with abnormally shaped embryos or differing in colour (Goodwillie & Knight, 2006); and (b) the number of well-developed seeds with visible and swollen embryos or germinated seeds. The rate of seed germination was calculated as the proportion of germinated seeds 40 days after sowing (stage of protocorm).

Linear mixed effects models were used to test whether seed viability and germination rate differed between the species and populations using the R 3.2.4 software and lme4 package (Bates et al., 2015). Because there were usually three sets of seed sown from one fruit, an individual identification number was set as a random effect. Before analysis, the log of the germination rate was taken and seed viability was transformed using Box Cox transformation to fit the assumptions of a normal distribution. Results were reported using the *sjPlot* package (Lüdecke, 2018). Seed germination ratios from experimental crossings were compared using the Kruskal–Wallis test and Dunn’s test of multiple comparisons.

## Results

### Levels and the determinants of reproductive success

Populations of both species differed in FRS between years (Table 2). Differences between populations of one species were sometimes larger than among distinct species. The highest fruiting among populations from the SPK over 3 years was noted in the SMOLF *P. bifolia* population (always almost 80% or more). Low fruiting in this region was observed in the POB1 *P. bifolia* and POB2 *P. chlorantha* populations in 2015 and 2017. In 2017, when seven populations from three regions were analysed, the highest FRS was noted in the SMOLF and SMOLM *P. bifolia* populations (without differences between meadow *vs*. forest) and in the forest *P. chlorantha* population (BF) from BNP, and the lowest FRS was in the *P. bifolia* populations (BC and POB1). The level of fruiting was not dependent on inflorescence flower position (U = 1.88, *p* = 0.06). Additive models of MRS and FRS outperformed models with species that interacted with variables (ΔBIC = 12.04 in FRS and ΔBIC = 11.29 in MRS). In particular for female flowers, models of RS explained the limited share of these trait variances for floral display and population density traits (Table 3). The overall FRS was not affected by the spatial structure, but it was mildly increased by floral display traits (Table 3).

**Table 2:** Spatio-temporal variation of floral display and reproductive success in *P. bifolia* and *P. chlorantha* populations in NE Poland.

*P.bifolia*													*P.chlorantha*													
	2015		2016		2017				Differences between years				2015			2016			2017				Differences between years			
	SMOL	POB1	SMOL	POB1	SMOL	POB1	POG	BC	SMOL	POB1	POG	BC	BON	LIN	POB2	BON	LIN	POB2	BON	LIN	POB2	BF	BON	LIN	POB2	BF
Plant height cm	39.8 ±7.3	36.2 ±7.2	37.0 ±4.8	31.0 ±6.6	36.9 ±8.3	30.3 ±8.2	50.1 ±9.8	44.0 ±8.6	ns	**	–	–	43.2 ±6.4	34.2 ±7.3	39.0 ±13.6	40.8 ±5.3	38.8 ±7.2	34.2 ±3.8	41.6 ±9.0	36.6 ±6.3	33.8 ±1.1	40.1 ±7.2	ns	**	ns	–
Differ. between pop	*		***		***								***			**			***							
Infl. lenght cm	10.6 ±3.0	8,7 ±2.6	7.6 ±1.9	7.0 ±1.4	9.5 ±2.9	7.1 ±2.6	12.7 ±4.1	9.9 ±3.1	***	**	–	–	11.6 ±2.7	8.4 ±2.9	10.7 ±5.7	9.3 ±2.3	8.6 ±2.9	7.9 ±1.8	10.0 ±3.8	9.3 ±2.6	7.0 ±2.8	11.4 ±4.0	***	ns	ns	–
Differ. between pop.	**		ns		***								***			ns			***							
Number of flowers	15.8 ±4.2	15.4 ±4.3	15.8 ±3.9	12.8 ±4.1	14.5 ±4.3	11.7 ±4.5	15.6 ±5.1	16.2 ±5.7	ns	***	–	–	14.0 ±3.0	15.1 ±4.2	11.1 ±6.0	13.1 ±2.5	14.2 ±4.3	14.2 ±3.4	9.9 ±3.8	10.9 ±4.2	9.0 ±2.8	14.8 ±5.0	***	***	ns	–
Differ. between pop.	ns		***		***								*			ns			***							
Pollinaria removal	77.5 ±20.0	50.7 ±27.7	78.9 ±16.0	66.6 ±25.2	80.0 ±25.5	56.9 ±36.7	85.9 ±18.2	86.8 ±20.4	ns	ns	–	–	53.3 ±28.1	92.1 ±6.6	65.9 ±24.4	67.1 ±18.1	87.4 ±12.9	67.3 ±12	78.9 ±24.3	95.2 ±11.6	83.5 ±10.7	87.3 ±14.8	***	**	ns	–
Differ. between pop.	***		**		***								***			***			***							
Flowers with both pollinaria removed MRS	83.9 ±17.0	76.7 ±29.8	75.5 ±19.0	67.2 ±27.7	96.7 ±8.1	80.7 ±26.1	94.9 ±6.8	98.0 ±5.5	***	ns	–	–	56.4 ±29.6	95.6 ±7.7	76.6 ±19.6	59.5 ±21.3	83.1 ±15.3	53.4 ±14.2	78.5 ±26.3	96.8 ±5.8	67.1 ±21.3	85.9 ±15.1	**	***	ns	–
Differ. between pop.	ns		ns		***								***			***			***							
Fruit set FRS	80.2 ±27.4	27.8 ±23.2	84.2 ±21.6	67.7 ±34.8	73.7 ±21.5	27.2 ±32.7	58.6 ±33.2	17.5 ±21.2	*	***	–	–	55.6 ±32.3	46.9 ±22.5	33.5 ±34.0	54.7 ±35.5	73.0 ±28.9	68.3 ±22.3	52.0 ±35.2	58.8 ±25.8	5.6 ±9.6	73.7 ±24.3	ns	**	**	–
Differ. between pop.	***		**		***								ns			ns			***							

**Note:**

* < 0.05, ** < 0.01, *** < 0.001; ns - non-significant.

**Table 3:** Results of generalized linear models with beta distribution explaining male and female reproductive success in 2015–2017 in Platantherans in the North-Eastern Poland with floral display and population density. Significant differences are given in bold.

	*Male reproductive success*			*Female reproductive success*		
*Predictors*	*Estimates*	*CI*	*p*	*Estimates*	*CI*	*p*
(Intercept, incl. *bifolia*)	0.53	[−0.05 to 1.12]	0.074	−1.44	[−2.13 to −0.75]	**<0.001**
Species [*chlorantha*]	0.28	[0.03–0.52]	**0.029**	0.50	[0.21–0.79]	**0.001**
Inflorescence height	0.01	[−0.04 to 0.05]	0.773	0.07	[0.01–0.12]	**0.016**
Number of flowers	−0.04	[−0.07 to −0.01]	**0.002**	0.04	[0.01–0.07]	**0.014**
Shoot height	0.04	[0.02–0.06]	**<0.001**	0.00	[−0.02 to 0.02]	0.883
Shoot density (kernel)	0.93	[−1.18 to 3.04]	0.387	−0.11	[−2.61 to 2.39]	0.931
Min. distance to neighbour	−0.02	[−0.06 to 0.02]	0.273	−0.03	[−0.08 to 0.02]	0.215
Mean distance to neighbour	−0.02	[−0.02 to −0.01]	**<0.001**	−0.01	[−0.01 to 0.00]	0.068
Observations	477			477		
R^2^	0.192			0.089		

MRS was significantly higher than FRS (Table 2). Among populations studied for 3 years, the highest MRS in each year was in the meadow LIN *P. chlorantha* population. In 2017, an extremely high pollen export was observed in two meadow populations—the LIN *P. chlorantha* (95.2%) and SMOLM *P. bifolia* (98.9%). Generally, in *P. bifolia*, more flowers had two pollinaria removed than in *P. chlorantha* (excluding the single population of each species). Temporary changes in MRS were observed in the BON and LIN *P. chlorantha* populations (Table 2). Pollen removal was greater in *P. chlorantha* and was affected both by the spatial population structure and floral display (Table 3). A denser population and higher shoots positively affected MRS, but the number of flowers had a negative effect on MRS. In each year, populations of both species differed in plant height, while temporary changes were noted only in POB1 and LIN populations (Table 2). The number of flowers per inflorescence varied significantly among populations, excluding *P. bifolia* in 2015 and *P. chlorantha* in 2016. Differences in the flower number between years were noted in POB1 and POG *P. bifolia* populations and three *P. chlorantha* populations (BON, LIN, and BF) (Table 2).

Stronger importance of floral display was noted when we compared fruiting and fruitless shoots in BC and POB1 populations with the lowest FRS. In both populations, fruitless shoots produced fewer flowers (*F*_*1*_ = 11.431, *p* = 0.001 and *F*_*1*_ = 9.373, *p* = 0.003) and had shorter inflorescences (*F*_*1*_ = 12.224, *p* = 0.0007 and *F*_*1*_ = 3.984, *p* = 0.05). In BON, there were fewer flowers on fruitless shoots than on those with fruits (*F*_*1*_ = 15.486, *p* = 0.0002).

We found a difference in MRS:FRS ratios and the highest disparity was observed in meadow populations of both species, especially in the BC *P. bifolia* population (MRS:FRS equalled five, Table 2). In the remaining populations, it ranged from 1 to 2.1 in different years.

#### Flower traits

Both species were represented by short- and long-spurred populations. Generally, the longer spurs (over 3 cm on average in each year) were observed in *P. chlorantha*, excluding LIN (Table S1). We also noted one long-spurred *P. bifolia* population (POG). The values of this trait varied substantially between populations of the two species, excluding *P. bifolia* in 2015 (Table S1). Temporal differences in spur length were noted only in the BF *P. chlorantha* population. Labellum length and width, size of spur entrance, distance between the viscidia, and pollinarium length were species-specific, and generally had larger values in *P. chlorantha* (Table S1). In BON and POB2 *P. chlorantha* populations, we found a relatively high proportion of individuals with overlapping values of some traits with those in the *P. bifolia* (Fig. S1). Among populations of both species, temporary variation was observed (Table S1).

The nectar column did not differ significantly between *P. chlorantha* populations(*F*_*1*_ = 1.436, *p* = 0.232), nor between *P. bifolia* (*F*_*1*_ = 0.974, *p* = 0.325) in both years. A temporary difference in the nectar column was found only in LIN (*F*_*1*_ = 15.371, *p* = 0.0001). In some populations and some years (five of 12 cases), the nectar column was positively correlated with the spur length (Table S1).

**Figure 1:**
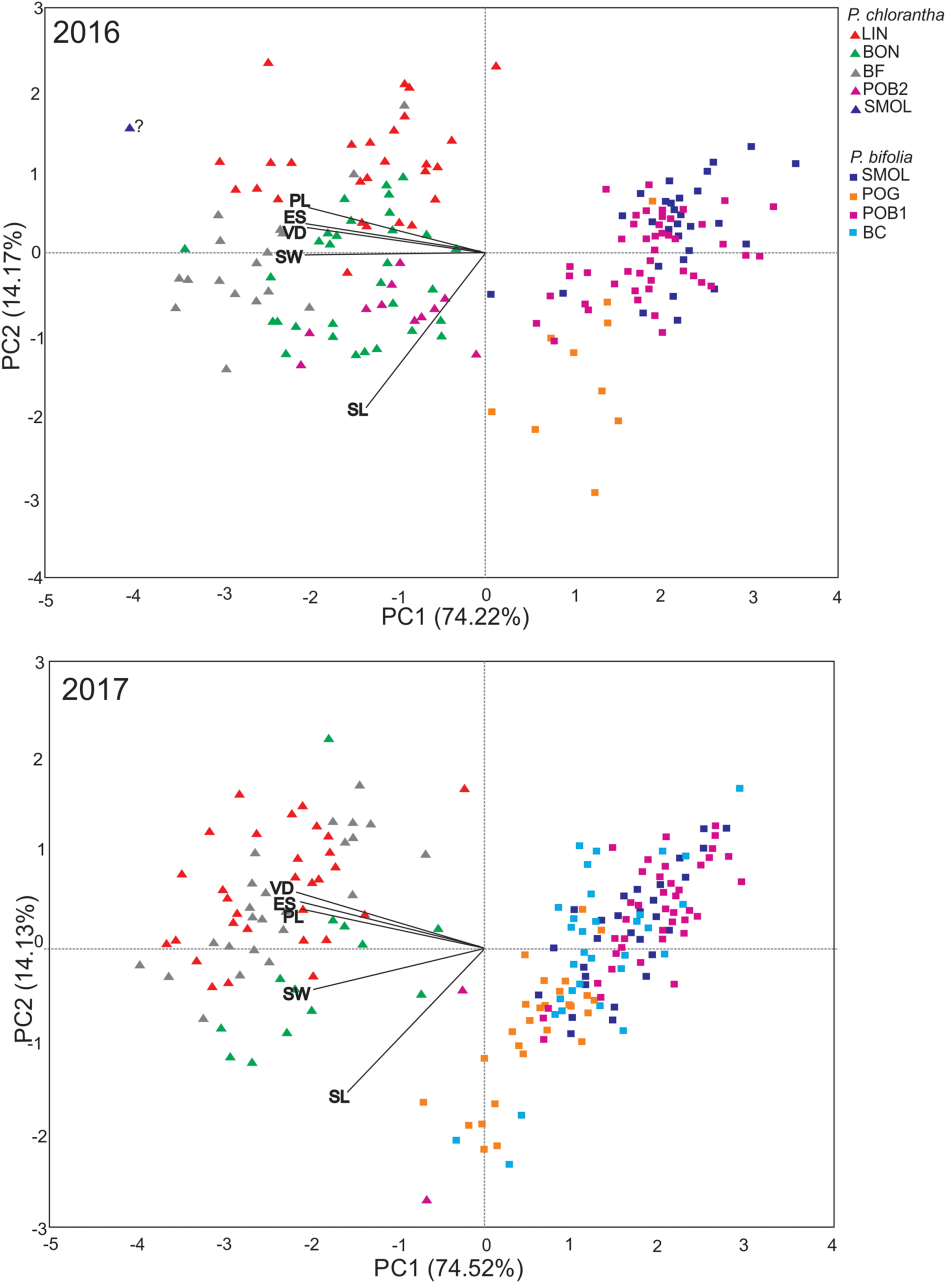
PCA analysis of floral traits in *P. bifolia* and *P. chlorantha* studied populations in 2016 and 2017. LL, labellum length; LW, labellum width; PL, pollinaria length; VD, distance between viscidia; ES, width of spur entrance.

PCA showed similar results for the 2 years and explained 89.39% and 88.65% of the variance along two main axes (Fig. 1). The first principal component encompassed 75.22% in 2016 and 74.52% in 2017 of the total variance. The flower traits clearly differentiated two species into separate clusters (Fig. 1). The cluster representing *P. bifolia* is more coherent than that of *P. chlorantha*. *P. chlorantha* populations form more discrete groups, especially the BON population, which was separated from the others (Fig. 1). The intermediate position between *P. bifolia* and *P. chlorantha* clusters occupied putative hybrids (from 1% to 7% of the populations). They exist primarily in mixed (POB1+POB2) and in BON *P. chlorantha* populations. In 2016, one potential hybrid in the SMOLF *P. bifolia* population with one *P. chlorantha* individual was found. PCA results suggest the presence of one intermediate in the BF and LIN in 2017. In 2016 (more shoots were observed in the BON and POB2 *P. chlorantha* populations), four such individuals were positioned on the PCA diagram closer to *P. bifolia* than to *P. chlorantha* individuals from the other or even the same population of this species (Fig. 1). Both individuals present in the POB2 in 2017 showed intermediate traits. Models of RS, including species only as an additive factor, outperformed those where there was interaction with the flower traits (by ΔBIC = 22.71 in males and 10.49 in females). The models explained slightly more of the MRS variance compared to the FRS variance (19.5% *vs*. 16%). Both MRS and FRS were affected by flower traits and were lower in *P. chlorantha* (Table 4). In both sexes, the RS increased with increase in the spur entrance width, but it decreased with the distance between viscidia. FRS decreased when the pollinarium length increased.

**Table 4:** Results of generalized linear models with beta distribution explaining male and female reproductive success in 2016–2017 in Platantherans in North-Eastern Poland with flower morphology traits. Significant differences are given in bold.

	*Male reproductive success*			*Female reproductive success*		
*Predictors*	*Estimates*	*CI*	*p*	*Estimates*	*CI*	*p*
(Intercept, incl. *bifolia*)	1.16	[0.80–2.80]	**<0.001**	1.16	[−0.02 to 2.35]	0.054
Species [*chlorantha*]	1.51	[1.21–1.81]	**<0.001**	0.96	[0.62 to −1.30]	**<0.001**
Spur length	−0.97	[−1.32 to −0.62]	**<0.001**	−1.23	[−1.65 to 0.82]	**<0.001**
Spur entrance	−0.16	[−0.34 to 0.01]	0.072	0.11	[−0.10 to 0.32]	0.301
Distance between viscidia	0.32	[0.07–0.57]	**0.014**	−0.43	[0.13–0.73]	**0.005**
Pollinarium length	−0.41	[−0.58 to −0.23]	**<0.001**	−0.40	[−0.59 to −0.20]	**<0.001**
Observations	237			247		
R^2^	0.195			0.164		

#### Moth assemblages

Moth assemblages showed wide variation in both *Platanthera* populations. The number of species ranged from six (POB1+POB2 mixed population, 2016) to 58 (LIN 2016 and POG 2017), and the number of captured individuals ranged from 6 (POB1+POB2, 2017) to 220 (POG 2017) (Table S4). The number of species that are known to be *P. bifolia* and *P. chlorantha* pollinators as well as their individual numbers was markedly lower (0–9 and 0–38, respectively) (Table S4). Pollinaria were found on only one *Cucullia umbratica* individual in the LIN *P. chlorantha* population. Most potential pollinators belonged to Noctuidae (Table S4). In some populations, they were represented only by a single species and individuals. Two species (*Diarsia mendica* and *Agrotis exclamationis*) were observed with a higher frequency. In populations in which moths were captured over two consecutive years, both the number of species that were known as pollinators and the individual numbers were markedly lower in 2017. Both the total number of moth species that were trapped and their individual numbers positively influenced MRS in 2017 (r = 0.83 and r = 0.75, respectively) but not FRS. The length of the moth proboscises was within the range that was reported in other studies (Table S4).

#### Pollen flow

**Figure 2:**
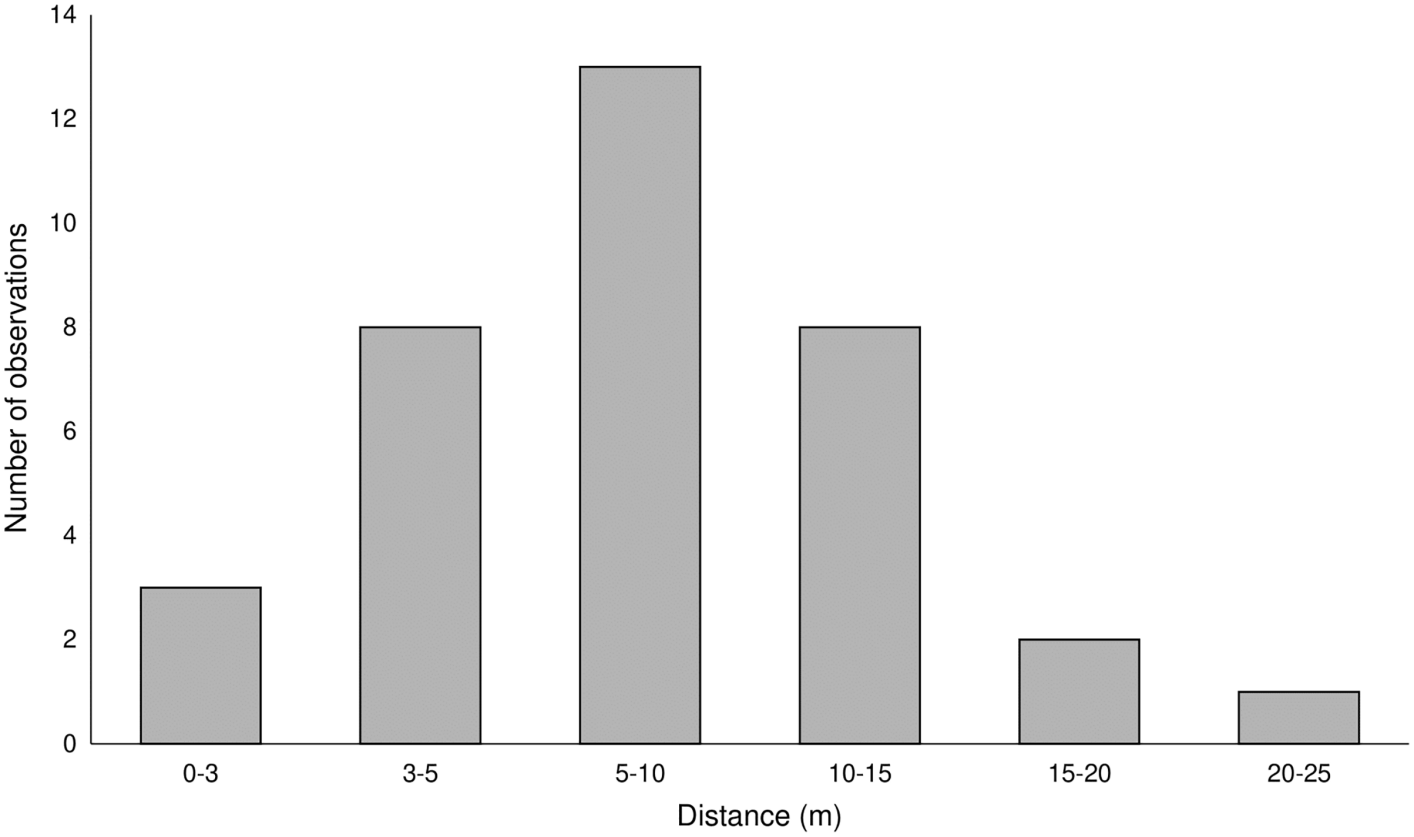
Distance of registered pollen flow observed between color-marked flowers of two *Platanthera* species.

Pollen with pigments was transferred up to 25 m (average 8.2 ± 4.83 m) from the donor plants (Fig. 2, Fig. S1) and was found on 102, 43, 14, and six stigmas in the LIN, SMOLF, BON, and POB1 populations, respectively. During one month visit, up to nine flowers on the inflorescence may be pollinated, but most frequently, one to three flowers were visited. Pollinator-mediated autogamy or geitonogamy were observed in 13.7%, 67.4%, 78.5%, and 100% of flowers with marked pollen deposited on the stigma in the LIN, SMOLF, BON, and POB1 populations, respectively. Pollen deposited on some stigmas was derived from different sources—in the LIN population we found two colours on six plants, while in the SMOLF population we observed two colours on one shoot (Fig. S1). In the first population, stigmas on one plant possessed pollen with three different pigments. In the SMOLF population, stigmas in seven *P. chlorantha* flowers (one individual among *P. bifolia*) received red coloured pollen from *P. bifolia*. All seven flowers developed into fruits.

### Compatibility experiments and seed viability and germination

**Figure 3:**
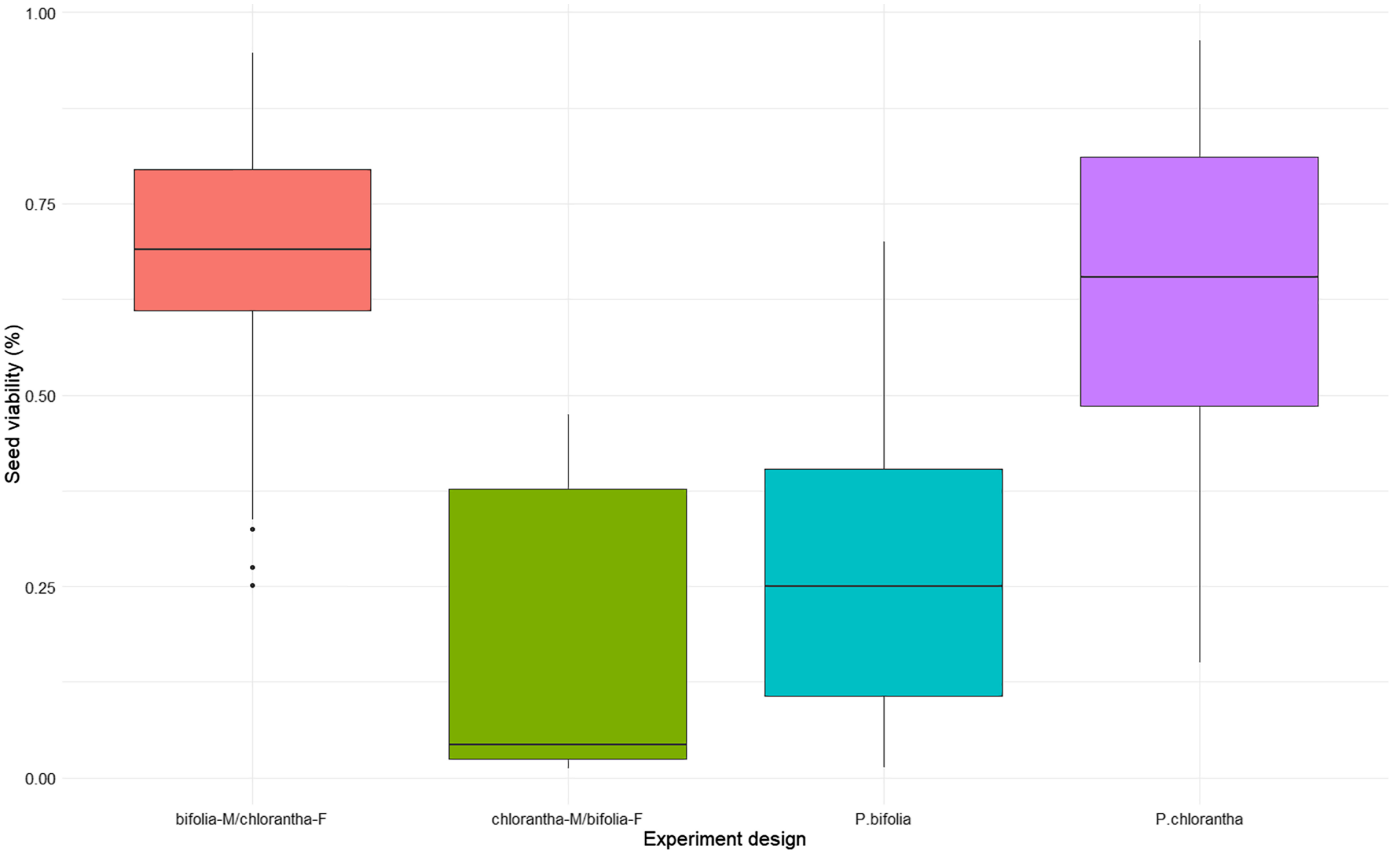
Comparison of seed viability in *Platanthera bifolia, P. chlorantha* and their crossings.

**Figure 4:**
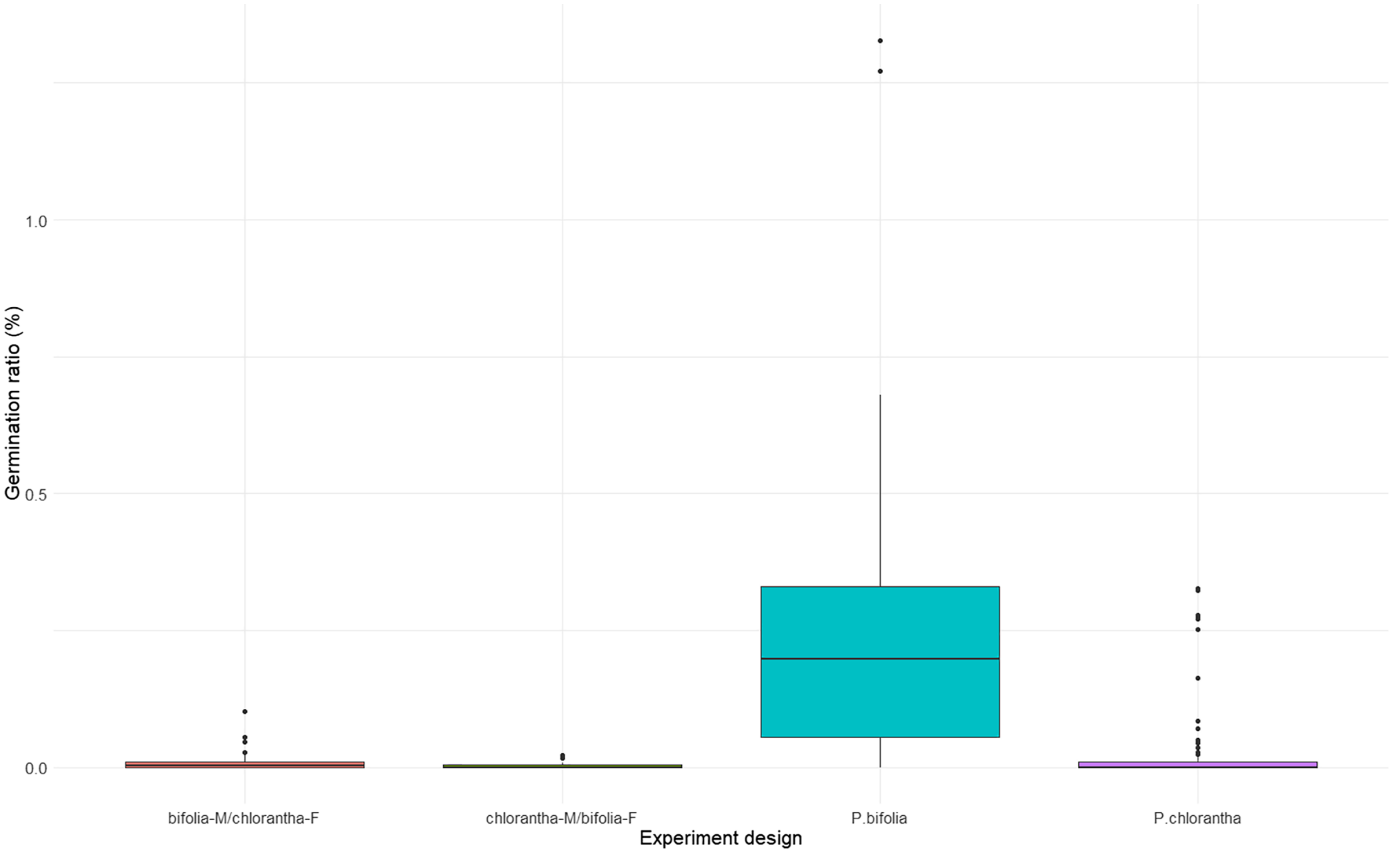
Comparison of seed germination rate in *Platanthera bifolia, P. chlorantha* and their crossings.

Seed viability differed between species and their crossings (Kruskal–Wallis, χ^2^ = 18.486, *p* = 0.00035), with *P. chlorantha* showing higher seed viability. It also differed between some populations (Fig. 3, Table S2). The seed germination rate was generally low, but it was clearly higher (*p* = 0.001) in both *P. bifolia* populations compared to any of the *P. chlorantha* populations (Fig. 4, Table S3).

In the crossing experiments, the germination rate differed between combinations of species and their crossings, incl. crossing direction (Kruskal–Wallis, χ^2^ = 20.184, *p* = 0.00015). A significantly higher germination rate was attributed to *P. bifolia* than to *P. chlorantha* seeds, which developed as a result of interspecies crosses (Fig. 4, Table 5). We did not find differences in the rate of seed germination between *P. chlorantha* seeds from natural pollination and seeds from interspecies crosses when the receiver was *P. chlorantha* and the donor was *P. bifolia*. A similar pattern of seed germination was observed in both years.

**Table 5:** Differences in seed viability and germination rate between *Platanthera bifolia, P. chlorantha* and their crossings tested with Dunn’s test of multiple comparisons. Significant differences are given in bold.

Comparison between species and hybrids		Seed viability		Germination rate	
Group1	Group2	Z	*p*	*Z*	*p*
*bifolia-M/chlorantha-F*	*chlorantha-M/bifolia-F*	1.300	0.233	1.541	0.185
*bifolia-M/chlorantha-F*	*bifolia*	−2.386	**0.034**	−2.461	**0.028**
*chlorantha-M/bifolia-F*	*bifolia*	−3.016	**0.008**	−3.317	**0.003**
*bifolia-M/chlorantha-F*	*chlorantha*	1.375	0.253	0.884	0.377
*chlorantha-M/bifolia-F*	*chlorantha*	−0.483	0.629	−1.074	0.339
*bifolia*	*chlorantha*	4.250	**0.0001**	3.829	**0.0008**

Interspecies cross-pollination showed that *bifolia*-M/*chlorantha*-F crossings had high seed viability and were comparable with *P. chlorantha*, while *bifolia*-M/*chlorantha*F crossings and *P. bifolia* showed the lowest seed variability (Fig. 3, Table 5). The germination rate, however, was the highest in *P. bifolia* and very low in *P. chlorantha* and both crossings (Fig. 4, Table 5). For the crossings, the germination rate was significantly higher in *bifolia*-M/*chlorantha*-F than *chlorantha*-M/*bifolia*-F crossings.

## Discussion

### Reproductive success and its determinants

Our results document a high level of RS of both Platantherans, excluding two *P. bifolia* populations. It is consistent with the rule that nectar-rewarding orchids are more successful in setting fruits than nectarless ones (Neiland & Wilcock, 1998; Tremblay et al., 2005). The RS in orchids is greatly differentiated and depends on many factors, one of which is floral display. Plants with larger inflorescences and more flowers better attract pollinators, which visit more flowers on larger inflorescences (Hodges, 1995; Maad, 2000; Grindeland, Sletvold & Ims, 2005; Vallius, Arminen & Salonen, 2006). If larger inflorescences are subject to factors that decrease their fitness (higher geitonogamy or intense herbivory), small inflorescences are favoured (Calvo, 1990; Vallius, Arminen & Salonen, 2006; Pellegrino, Bellusci & Musacchio, 2010). In our studies, the number of flowers was more important for RS than plant height, although in the BON *P. chlorantha* population with the lowest shoot density, the number of fruits depended on shoot height each year. The MRS was less dependent on the floral display, which contrasts with Bateman’s principle (the male function hypothesis) that selection of the flower number should be stronger through male compared to female function because fruiting is resource-limited (Burd & Callahan, 2000). Maad & Alexandersson (2004) found sex-differentiated selection of flower number in *P. bifolia*, but only through male function under drought conditions. The influence of pollinators on inflorescence height may be stronger in taller than in shorter vegetation (Ågren, Fortunel & Ehrlén, 2006; Sletvold, Grindeland & Ågren, 2013; Boberg et al., 2014), but our results do not support this finding.

Phenotypic selection depends on the mutual match between pollinator and flower morphology (Moré et al., 2012). Therefore, flower traits play a crucial role in attracting pollinators. In long-spurred plants, among the most important flower traits influencing RS is spur length (Inoue, 1986; Nilsson, 1988; Maad, 2000; Alexandersson & Johnson, 2002; Little, Dieringer & Romano, 2005; Whittall & Hodges, 2007; Boberg & Ågren, 2009; Boberg et al., 2014; Trunschke, Sletvold & Ågren, 2018). In our studies, the fruit number increased with the increase in spur length in *P. bifolia*. This is consistent with the expectation that selection on longer spurs occurs if the spur is too short to match the primary pollinator proboscis length (Maad, 2000; Boberg & Ågren, 2009; Boberg et al., 2014). In *P. chlorantha*, depending on the year, the spur length increased or decreased FRS. This may be caused by a change in the main pollinator between years. Nilsson (1988) noted the effect of selection on spur length when the spur is longer than the moths’ proboscis. A negative relationship between RS parameters and the distance between viscidia and the pollinarium length suggest mismatch between flower structure and pollinators. The weak correlations between flower traits and RS could be also explained by Boberg & Ågren (2009)’s suggestion that the flower properties might be less important in plants pollinated by nocturnal insects. It can also indicate that plant-pollinator interactions are more complicated than expected, and RS is the effect of multiple factors acting together. The cause of weak or a lack of such relationships may be the small population size. A stronger influence of floral display and flower traits was noted when we pooled the data and compared fruitless shoots (which were shorter, had shorter spikes and fewer flowers) with those producing fruits.

RS in nectariferous plants is greatly affected by nectar quantity and quality (Duffy & Stout, 2008; Willmer, 2011; Gijbels, Van den Ende & Honnay, 2014; Gijbels et al., 2015; Gijbels, Van den Ende & Honnay, 2015; Brzosko & Bajguz, 2019). Higher nectar availability promotes longer visitation in single flowers and all the inflorescences (Hodges, 1995; Maad & Reinhammar, 2004), and in effect increases selfing (de Jong, Waser & Klinkhamer, 1993; Jersakova & Johnson, 2006). Similarly to Ackerman, Rodriguez-Robles & Melendez (1994) and Duffy & Stout (2008), we found a minimal effect of nectar level on RS, suggesting that the nectar quantity offered by orchids in the populations studied is sufficient to attract pollinators.

Viability of seeds from natural pollination, although varied between populations, was generally low and may be explained by the realised mating system. A pollen-tracking experiment showed that autogamy or geitonogamy are important for RS of both species. Selfing may increase fruiting, but can decrease seeds viability and cause inbreeding depression (Grindeland, Sletvold & Ims, 2005; Duffy & Stout, 2008), which is higher in rewarding plants (Sletvold et al., 2012). This effect could be elevated due to the small population sizes, which cause an increase in the selfing or mating between relatives. More frequent geitonogamy in small rather than large *P. bifolia* and *P. leucophaea* populations was observed by Maad & Reinhammar (2004) and Wallace (2002). Lower seed quality as result of selfing was found by Tałałaj et al. (2017) in *P. bifolia* and Metsare et al. (2015) in *P. chlorantha*. Generally, the populations studied occupied small areas with plants growing in close vicinity. Thus, for many generations, gene exchange took place between the same pool of individuals—pollen was transported mainly for short distances, and seeds are dispersed in close vicinity to the mother plants (Brzosko et al., 2017). All these circumstances promote selfing and crosses between relatives. Such an explanation of low seeds viability due to the above-mentioned causes confirm studies on genetic variation conducted in the same populations (Brzosko et al., 2009; Brzosko & Wróblewska, 2013).

### Spatial aspects of reproductive success

Plant-pollinator interactions and reproductive processes may depend on spatial distribution of resources in the population (*e.g*. nectar reward). It can influence pollinator activity and consequently affect the plant mating system and RS in different ways (Internicola et al., 2006; Juillet et al., 2007; Brys, Jacquemyn & Hermy, 2008; Duffy & Stout, 2008). According to the optimal foraging theory, the visitation rate is higher in dense patches, but in isolated plants, insects visit more flowers (Pyke, 1982). Duffy & Stout (2008) observed a lower visitation rate in denser patches of *Spiranthes romanzoffiana* due to competition for pollinators, while Grindeland, Sletvold & Ims (2005) did not find any effects caused by plant density. We noted that MRS, but not FRS, was density dependent. Higher MRS in denser patches suggests a higher moth visitation rate in these places, but the lack of correlation between shoot density and FRS may indicate that these visitors were not always pollinators. Despite the lack of density influence on FRS, we found a differentiated spatial pattern of fruiting in populations. In the POB1 and BC *P. bifolia* and the BON *P. chlorantha* populations, we noted a high proportion of fruitless shoots and low MRS. A possible cause for the high number of unvisited plants in the BON *P. chlorantha* population could be the low shoot density. Single shoots usually grow over long distances, causing low floral display, and thus are less visible for pollinators. This effect may be stronger in situations with a pollinator deficiency. However, *P. chlorantha* is almost exclusively the one species that flowers during this time and one of the tallest on the forest floor. For this reason, its visibility should be appropriate. In the BC *P. bifolia* population, a high proportion of fruitless shoots could be explained through competition for pollinators and/or their deficiency, as the density of both *P. bifolia* and co-flowering plants was high in this location.

We found that, in many cases, the number of pollinaria removed from the Platantherans flowers was much higher than the number of pollinated flowers and consequently the fruit set. Low pollination efficiency in the populations of other nectariferous species (*Listera ovata*) was found by Brys, Jacquemyn & Hermy (2008). According to Tremblay et al. (2005), in nectariferous temperate orchids, about 51.9% of pollinia were removed, while 41.8% of flowers set fruits. Maad (2000) noted lower MRS than FRS in *P. bifolia* and *P. chlorantha* populations. Our results show an opposite pattern than Maad’s (2000) observations in most populations, especially in BC and POB, where MRS was few times greater than FRS. The imbalance between MRS and FRS could be explained by the low efficiency of accidental visitors, which remove pollinaria but are not able to transfer them onto the stigmas due to a mismatch in partner morphology (Bateman, James & Rudall, 2012). Another explanation for this imbalance is high pollen discounting; the highest MRS:FRS ratio was found in meadow populations more exposed to wind. We observed pollinaria on neighbouring plants similarly to Friesen & Westwood (2013). Pollen discounting is also high when pollinators longer penetrate one inflorescence. Such behaviour is known for nectariferous plants (Vallius, Arminen & Salonen, 2006), and our experiment with pollen tracking confirms this finding.

### Moth assemblages

Plant species evolution is driven by many factors that have different roles in space and time. One of the most important evolutionary mechanisms is plant–pollinator interactions, which are reflected in the level of RS (Cozzolino & Widmer, 2005; Tremblay et al., 2005). Both Platantherans may share main pollinators (*Deilephila* species and *Hyloicus pinastri*; Nilsson, 1983), although more recent studies show differences in the main pollinators, which depend on the location in the geographical range and plant communities (Maad, 2000; Boberg et al., 2014; Sexton, 2014; Esposito, Merckx & Tyteca, 2017; Mõtlep et al., 2018). Noctuids pollinate mainly *P. chlorantha*, while sphingids *P. bifolia*, although the short-spurred flowers of the last species are also pollinated by noctuids (Nilsson, 1983). Common visitors of *P. bifolia* in Sweden were *Deilephila porcellus*, *D. elpenor*, *Hyloicus pinastri*, *Sphinx ligustri*, and *Hyles gallii* (Nilsson, 1983, 1988), but in central Sweden it was pollinated almost exclusively by *H*. *pinastri* (Maad, 2000). In Estonia, *P. bifolia* is mainly pollinated by *H. pinastri* and *S. ligustri*, while *P. chlorantha* by Noctuidae (Mõtlep et al., 2018). Boberg et al. (2014) found a pollinator shift in Scandinavian *P. bifolia* populations, and depending on flower traits, habitat and the geographical location, the main pollinators were *H. pinastri*, *S. ligustri*, *D. porcellus* or *Entephria caesiata*. We found an important part of the whole set of known pollinators of both species, but pollinaria were found on only one *Cucullia umbratica* in the *P. chlorantha* LIN population (the main pollinator in Belgium; Esposito, Merckx & Tyteca, 2017). An important pollinator, *D. porcellus*, was absent in our studies, and only one individual of another important pollinator, the long-tongued *S. ligustri*, was found in the SMOLF *P. bifolia* population. In other studies, despite fruiting, pollinators have never been observed (Pérez-Hérnandez et al., 2011; Fox et al., 2015) or noted sporadically with a frequency that does not reflect the level of RS (Moré et al., 2012; Steen & Mundal, 2013; Sexton, 2014; Esposito, Merckx & Tyteca, 2017; Mõtlep et al., 2018). Correlation between the MRS and the number of moth species and their individuals suggests that moths that are not known as pollinators or those that play a minor role in other regions might enhance RS. Potentially, two noctuids (*Agrotis exclamationis* and *Diarsia mendica*), observed with high numbers in studied populations could also serve as pollinators. A relatively high RS compared to a low frequency of potential pollinators could be explained through their higher abundance on other nights. The similar fruit set in the bottom and upper parts of inflorescence indicates that pollinators are active during the entire flowering time. The low RS in some populations may suggest pollinator deficiency. This is consistent with the finding that pollen limitation due to pollinator deficiency is common in orchids (Inoue, 1985; Johnson & Bond, 1997; Maad & Alexandersson, 2004; Tremblay et al., 2005; Vallius & Salonen, 2006; Boberg et al., 2014; Zhou et al., 2016). The low number of fruiting shoots in the BON *P. chlorantha* and the BC and POB1 *P. bifolia* populations in 2017 (20–44.1%), together with a lower moth diversity and numbers also suggest pollinator deficiency. Mõtlep et al. (2018) found that pollinator abundance affects the fruit set of *P. bifolia*, but not *P. chlorantha*.

Our results support the finding that pollinators change according to geographic location and habitat (Boberg et al., 2014; Esposito, Merckx & Tyteca, 2017). Most potential pollinators recorded belonged to Noctuidae and were present in all populations, while sphingids were noted in four populations. Taking into account the distances between the spur entrance and nectar levels and the proboscis length of noctuids (5–21 mm; Boberg et al., 2014; Sexton, 2014), we can recognise many of them as pollinators. Even taking into consideration that the flowers in *P. bifolia* POG population have the longest spurs with the highest nectar columns, moths with shorter proboscises might try to reach the drops from the spur wall and pollinate the flower. The correlation between spur length and the nectar column in BON and BF *P. chlorantha* and BC *P. bifolia* populations suggests flower adaptation to pollinators with a longer proboscis. This could explain the case of the BON population (long-spurred, but with low nectar level), without long-tongued moths, wherein despite the high number of potential pollinators, a lower fruiting was noted. Boberg et al. (2014) found that short-spurred *P. bifolia* in grasslands was pollinated mainly by the short-proboscis *D. porcellus*, while long-spurred in woodlands by the long-proboscis *S. ligustri*. Nilsson (1983) showed that both moths with long and short tongues may compete for nectar.

The pollen-tracking experiment documented the activity of pollinators. Pollinia were transported up to 25 m (average 8.2 m). Up to nine flowers per inflorescence were visited by one moth, which confirms that on inflorescences of nectariferous species, pollinators stay longer. On some stigmas, massulaes from different individuals were deposited, which indicates that more than one pollinator can visit a single flower. We also found that pollinator-mediated selfing in both species increased the fruiting (auto- or geitonogamy we observed in 13.7%, 67.4%, 78.5%, and 100% of flowers on inflorescences in different populations).

### Are reproductive isolation mechanisms enough for maintaining species integrity?

*P. bifolia* and *P. chlorantha* can produce hybrids (Nilsson, 1983; Maad & Nilsson, 2004; Bateman, James & Rudall, 2012; Esposito et al., 2018; Mõtlep et al., 2021). Our PCA analysis demonstrated the presence of intermediate morphotypes, localised mainly in mixed POB1+POB2 and in the BON *P. chlorantha* populations. Although we did not conduct genetic analyses, results of Mõtlep et al. (2021), who found that morphological traits well correlated with molecular traits, could suggest a high probability that the intermediate morphotypes are hybrids. The presence of putative hybrids in BON could be explained by mating between *P. chlorantha* from this population and *P. bifolia* individuals which we observed in 2005 in a neighbouring meadow, but later disappeared. One plant with intermediate traits is visible on the PCA diagram in 2016 in the SMOLF *P. bifolia* population, with a single *P. chlorantha*. Similarly to other studies (*e.g*., Nilsson, 1983; Esposito et al., 2018; Mõtlep et al., 2021) we found that intermediates are rare (1–7% of a particular population). Their number is probably higher because intermediates found in different years are rarely the same individuals (due to dormancy; Brzosko, 2003). In 2015, in the BON population, we observed more plants with intermediate flowers. They had pollinaria distanced as in *P. bifolia*, but a wide spur entrance and large stigma as in *P. chlorantha*. Results of experiments with interspecies crosses suggest a high potential for hybridisation. Seeds developed from interspecies crosses were viable and able to germinate, but with different frequency, which is in contrast to Mõtlep et al. (2021), who found no differences both in viable seeds and germination rate after interspecies crosses. In our studies, when *P. bifolia* was a receiver of *P. chlorantha* pollen, seeds were less viable than those from crosses in the opposite direction. Germination of both species was lower than seed viability, but the *P. bifolia* seeds germinated with higher frequency, which could be explained by a distinct mechanism controlling the germination of two species. However, germination was independent of the cross direction, which may suggest that postzygotic barriers act at different developmental phases. Lower seed viability from interspecies crosses with *P. chlorantha* as a pollen donor compared to the germination level of seeds produced naturally in *P. bifolia* may also suggest the presence of post-pollination isolation barriers. Spontaneous interspecies crossing was only noted in the SMOLF population, where flowers on only one *P. chlorantha* were pollinated by coloured *P. bifolia* pollen, and all of them set fruits with viable seeds. It confirms the Nilsson (1985) and Mõtlep et al. (2021) observations that pollen transfer always takes place from *P. bifolia* to *P. chlorantha*. This also shows that the same moths can pollinate both species. Nilsson (1992) found that the intermediate column alters interactions with moths, and consequently RS decreases. We confirm this finding; in 93% of cases, FRS and MRS of intermediates was lower. Our results could also suggest that intermediates are characterised by worse survival, because in populations with the highest number of intermediates, we observed a drastic decrease in individuals from year to year.

## Conclusions

Our results highlight the importance of investigations of reproductive strategies and their determinants in different parts of species’ geographical range for better understanding of the mechanisms and processes crucial for species evolution. This is confirmed by spatial variation of the FRS and MRS in *P. bifolia* and *P. chlorantha* and the differentiated role of factors influencing RS in populations studied, as well as by results of studies conducted in other European populations. We found that reproductive processes are rather site- than species-dependent. Variation of RS among populations suggest that adaptations to changing conditions of local environment and pollinator assemblages are in fact ongoing and crucial for population persistence. In our studies, the MRS was affected both by floral display traits and population spatial structure, while FRS depended only on floral display. On the whole, the flower traits had a weak influence on RS. A significantly higher level of the MRS over FRS suggests pollen discounting, especially in meadow populations, caused by neighbouring plants or a mismatch between morphology of visitors and flower structure. Finally, we found MRS correlated with diversity and the number of moths. This may suggest that moths that are not known as Platantherans pollinators might enhance RS in populations from NE Poland. Also, through an experiment with pollen tracking, we revealed the (small) spatial scale at which pollinators operate, importance of pollinator-mediated selfing for the overall RS, as well as that spontaneous interspecies crossing of Platantherans takes place in natural conditions.

We found that seeds that developed from interspecies crossing were viable and able to germinate. This result together with observations of spontaneous interspecies crosses in the field suggests that both pre- and postzygotic reproductive barriers are not complete and do not prevent hybrid production. These incomplete barriers have greater evolutionary significance in mixed populations where putative hybrids were observed. Thus, dilution of species integrity is more probable in places where Platantherans occur in sympatry. Overall, this suggests the importance of spatial isolation as a reproductive barrier, confirming the results of our experiment with pollen tracking.

## Supplemental Information

10.7717/peerj.13362/supp-1 Supplemental Information 1 Pollen flow experiments with color-marked shoots of two Platantherans species in three populations A) SMOL, B) LIN and C) BON.

10.7717/peerj.13362/supp-2 Supplemental Information 2 Spatio-temporal variation of flower traits in *P. bifolia* and *P. chlorantha* populations in NE Poland.

10.7717/peerj.13362/supp-3 Supplemental Information 3 Linear mixed effects models explaining seed viability in two species *Platanthera bifolia* and *P. chlorantha* and their different populations (see Tab. 1 and Tab. 2 in full article for populations description).

10.7717/peerj.13362/supp-4 Supplemental Information 4 Linear mixed effects models explaining germination rate in two species *Platanthera bifolia* and *P. chlorantha* and their different populations (see Tab. 1 and Tab. 2 in full article for populations description).

10.7717/peerj.13362/supp-5 Supplemental Information 5 The number of insect species and individuals within family collected in twelve populations in north-eastern Poland.

## Additional Information and Declarations

### Field Study Permissions

The following information was supplied relating to field study approvals (*i.e*., approving body and any reference numbers): Field experiments were approved by the Environmental Ministry of Poland (decision numbers: DOP-WPN.286.53.2017MŚ). Regional Director of Environmental Protection (decision numbers: WPN.6400.40.2016.MW, WPN.6400.15.2017.MR).

### Data Availability

The following information was supplied regarding data availability: Raw data used in this article are available at Mendeley: Mirski, Paweł; Brzosko, Emilia; Jermakowicz, Edyta (2021), “*Platanthera bifolia* and *Platanthera chlorantha* dataset, NE Poland 2015-2017”, Mendeley Data, V1, DOI 10.17632/2hgbv623wd.1.
