# Supplementary figures and images for: Pollen transfer and patterns of reproductive success in pure and mixed populations of nectariferous Platanthera bifolia and P. chlorantha (Orchidaceae)
Source: PeerJ. 2022 Jun 13;10:e13362. doi: 10.7717/peerj.13362 (PMC9202541; doi:10.7717/peerj.13362)

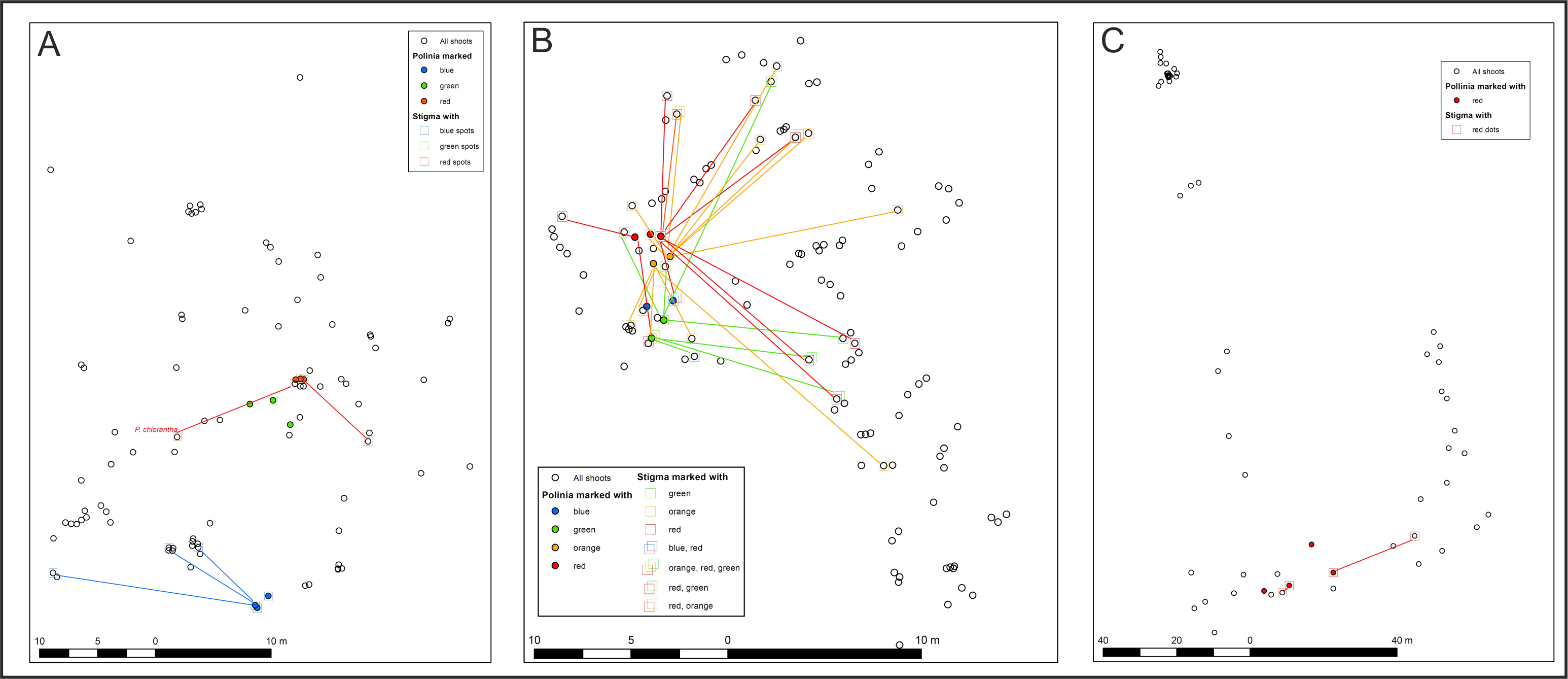

Supplement: Supplemental Information 1 [file peerj-10-13362-s001.png]
