# Supplementary material for: Pollen transfer and patterns of reproductive success in pure and mixed populations of nectariferous Platanthera bifolia and P. chlorantha (Orchidaceae)
Source: PeerJ. 2022 Jun 13;10:e13362. doi: 10.7717/peerj.13362 (PMC9202541; doi:10.7717/peerj.13362)
Supplement: Supplemental Information 2 [file peerj-10-13362-s002.docx]

| *Platanthera bifolia* | | | | | | | | | | | | | | *Platanthera chlorantha* | | | | | | | | | | | | | | |
| --- | --- | --- | --- | --- | --- | --- | --- | --- | --- | --- | --- | --- | --- | --- | --- | --- | --- | --- | --- | --- | --- | --- | --- | --- | --- | --- | --- | --- |
|  | 2015 | | 2016 | | | 2017 | | | | Differences between years | | | | 2015 | | | 2016 | | | | 2017 | | | | Differences between years | | | |
|  | SMOL | POB1 | SMOL | POB1 | POG | SMOL | POB1 | POG | BC | SMOL | POB1 | POG | BC | BON | LIN | POB2 | BON | LIN | POB2 | BF | BON | LIN | POB2 | BF | BON | LIN | POB2 | BF |
| Spur length | 26.80 ±3.22 | 26.31 ±3.27 | 24.50  ±2.88 | 24.84  ±3.33 | 32.90  ±3.56 | 25.28  ±3.15 | 25.09  ±3.29 | 33.67  ±3.58 | 27.46  ±4.61 | ns | ns | ns | - | 35.29  ±2.71 | 27.43  ±3.15 | 35.92  ±2.93 | 34.58  ±2.78 | 26.31  ±2.94 | 35.12  ±6.15 | 33.47  ±3.18 | 33.53  ±4.38 | 26.99  ±2.82 |  | 30.77  ±3.51 | ns | ns | - | *** |
| Differ. between pop | ns | | *** | | | *** | | | |  | | | | *** | | | *** | | | | *** | | | |  | | | |
| Labellum length |  |  | 8.69  ±1.49 | 9.59  ±1.58 | 12.96  ±2.07 | 10.25  ±1.54 | 9.89  ±1.56 | 12.94  ±1.69 | 10.58  ±1.88 | *** | ns | ns | - | - |  |  | 12.33  ±1.47 | 9.76  ±1.85 | 12.36  ±1.97 | 13.01  ±1.69 | 13.43  ±2.47 | 12.41  ±1.48 | 12.87 | 12.55  ±1.62 | * | *** | - | ns |
| Differ. between pop. | - | | *** | | | *** | | | |  | | | | - | | | *** | | | | ns | | | |  | | | |
| Labellum width |  |  | 1.78  ±0.27 | 1.84  ±0.29 | 2.07  ±0.42 | 2.17  ±0.29 | 1.99  ±0.47 | 2.39  ±0.34 | 2.21  ±0.26 | *** | P=0.06 | ** | - | - |  |  | 2.99  ±0.31 | 2.80  ±0.28 | 2.71  ±0.35 | 3.07  ±0.37 | 2.88  ±0.37 | 2.98  ±0.45 | 2.31 | 2.96  ±0.37 | ns | ** | - | ns |
| Differ. between pop. | - | | ** | | | *** | | | |  | | | | - | | | *** | | | | ns | | | |  | | | |
| Spur entrance | 0.72  ±0.25 | 0.74  ±0.19 | 0.51  ±0.16 | 0.55  ±0.16 | 0.59  ±0.15 | 0.55  ±0.09 | 0.51  ±0.13 | 0.61  ±0.14 | 0.59  ±0.15 | ns | ns | ns | - | 1.80  ±0.29 | 1.82  ±0.25 | 1.51  ±0.20 | 1.29  ±0.18 | 1.35  ±0.22 | 1.23  ±0.44 | 1.25  ±0.18 | 1.42  ±0.19 | 1.56  ±0.24 | 0.88 | 1.21  ±0.15 | * | *** | - | ns |
| Differ. between pop. | ns | | ns | | | ** | | | |  | | | | *** | | | ns | | | | *** | | | |  | | | |
| Distance between viscidia | - | - | 0.56  ±0.17 | 0.69  ±0.17 | 1.01  ±0.21 | 0.62  ±0.21 | 0.56  ±0.15 | 0.74  ±0.11 | 0.55  ±0.14 | ns | *** | *** | - | - | - | - | 2.15  ±0.57 | 2.46  ±0.63 | 1.63  ±0.62 | 3.47  ±0.69 | 2.53  ±0.77 | 2.99  ±0.55 | 1.27 | 3.30  ±0.36 | * | ** | - | ns |
| Differ. between pop. | - | | *** | | | *** | | | |  | | | | - | | | *** | | | | *** | | | |  | | | |
| Pollinaria length | - | - | 2.16  ±0.30 | 2.19  ±0.34 | 2.31  ±0.47 | 2.09  ±0.24 | 2.05  ±0.26 | 2.48  ±0.29 | 2.33  ±0.24 | ns | * | ns | - | - |  |  | 3.19  ±0.26 | 3.57  ±0.35 | 2.66  ±0.34 | 3.71  ±0.38 | 3.28  ±0.31 | 3.65  ±0.29 | 3.02 | 3.90  ±0.44 | ns | ns | - | ns |
| Differ. between pop. | - | | ns | | | *** | | | |  | | | | - | | | *** | | | | *** | | | |  | | | |
| Nectar | 7.20  ±3.94 | 8.55  ±3.99 | - | - | - | 8.69  ±4.49 | 7.61  ±3.53 | 15.94  ±5.05 | 5.79  ±5.42 | ns | ns | - | - | 9.72  ±3.61 | 5.49  ±2.79 | 14.31  ±5.29 | - | - | - | - | 9.80  ±5.55 | 7.87  ±3.04 | 5.19 | 4.49  ±3.09 | ns | *** | - | - |
| Differ. between pop. | ns | | - | | | *** | | | |  | | | | *** | | | - | | | | *** | | | |  | | | |
