# Supplementary material for: Pollen transfer and patterns of reproductive success in pure and mixed populations of nectariferous Platanthera bifolia and P. chlorantha (Orchidaceae)
Source: PeerJ. 2022 Jun 13;10:e13362. doi: 10.7717/peerj.13362 (PMC9202541; doi:10.7717/peerj.13362)
Supplement: Supplemental Information 3 [file peerj-10-13362-s003.docx]

|  | **Seed viability** | | |
| --- | --- | --- | --- |
| *Predictors* | *Estimates* | *CI* | *p* |
| (Intercept, incl. *P. bifolia*) | -0.35 | -0.41 – -0.29 | **<0.001** |
| *P. chlorantha* | 0.09 | 0.04 – 0.15 | **0.001** |
| LIN | -0.06 | -0.50 – 0.38 | 0.794 |
| POB | -0.47 | -0.55 – -0.40 | **<0.001** |
| **RandomEffects** | | | |
| σ^2^ | 0.00 | | |
| τ_00_ _Ind_ | 0.05 | | |
| ICC _Ind_ | 0.92 | | |
| Observations | 256 | | |
| Marginal R^2^ / Conditional R^2^ | 0.539 / 0.965 | | |
