# Supplementary material for: Pollen transfer and patterns of reproductive success in pure and mixed populations of nectariferous Platanthera bifolia and P. chlorantha (Orchidaceae)
Source: PeerJ. 2022 Jun 13;10:e13362. doi: 10.7717/peerj.13362 (PMC9202541; doi:10.7717/peerj.13362)
Supplement: Supplemental Information 4 [file peerj-10-13362-s004.docx]

|  | **log(germination rate)** | | |
| --- | --- | --- | --- |
| *Predictors* | *Estimates* | *CI* | *p* |
| (Intercept, *incl. P. bifolia*) | 1.19 | 0.76 – 1.61 | **<0.001** |
| *P. chlorantha* | -0.60 | -1.01 – -0.20 | **0.003** |
| LIN | -0.36 | -0.60 – -0.12 | **0.004** |
| POB | 0.81 | 0.42 – 1.20 | **<0.001** |
| SMOL | 1.21 | 0.76 – 1.66 | **<0.001** |
| Random Effects | | | |
| σ2 | 0.53 | | |
| τ_00_ individual | 0.50 | | |
| ICC individual | 0.48 | | |
| Observations | 619 | | |
| Marginal R^2^ / Conditional R^2^ | 0.421 / 0.702 | | |
