# Supplementary material for: Pollen transfer and patterns of reproductive success in pure and mixed populations of nectariferous Platanthera bifolia and P. chlorantha (Orchidaceae)
Source: PeerJ. 2022 Jun 13;10:e13362. doi: 10.7717/peerj.13362 (PMC9202541; doi:10.7717/peerj.13362)
Supplement: Supplemental Information 5 [file peerj-10-13362-s005.docx]

| **Family** | **Species** | LIN_16 | LIN_17 | BON_16 | BON_17 | POB_16 | POB_17 | SMOL_16 | SMOL_17 | POG_16 | POG_17 | BF_17 | BC_17 |
| --- | --- | --- | --- | --- | --- | --- | --- | --- | --- | --- | --- | --- | --- |
| Arctidae | *Diacrysiasannio* |  |  |  |  | 3 | 1 | 1 | 1 |  |  | 1 |  |
|  | *Atolmisrubricollis* |  |  |  |  |  |  | 1 |  |  |  |  |  |
|  | *Miltochristaminiata* |  |  |  |  |  |  |  |  | 1 |  | 3 |  |
|  | *Cybosiamesomella* |  |  |  |  |  |  |  |  |  |  | 3 |  |
|  | *Spilosomalubricipeda* | 1 | 5 | 10 | 2 | 5 |  | 6 | 10 | 18 | 1 | 2 | 24 |
|  | *Spilosomaurticae* |  |  |  |  |  |  |  |  |  |  |  |  |
|  | *Spilarctialutea* |  |  | 3 | 9 | 1 |  | 5 | 11 | 6 |  | 21 | 16 |
| Cossidae | *Phragmitaeciacastanea* |  |  |  |  |  |  |  |  | 2 | 4 |  |  |
| Drepanidae | *Tetheaor* |  |  | 1 |  |  |  |  |  |  |  |  |  |
|  | *Drepanafalcataria* |  |  | 1 |  |  |  |  |  |  | 1 |  |  |
|  | *Drepanacurvulata* |  |  |  |  |  |  |  |  | 1 |  |  |  |
|  | *Habrosynepyritoides* |  |  |  |  |  |  |  |  |  |  | 1 | 3 |
| Geometridae | *Calospilossylvata* |  |  |  |  |  |  |  |  | 1 |  | 5 | 1 |
|  | *Lomaspilismarginata* |  |  | 1 |  | 1 |  | 2 | 1 |  |  |  | 1 |
|  | *Macaria notata* |  |  | 1 |  |  |  |  | 2 |  |  |  |  |
|  | *Macarialiturata* |  |  |  |  | 3 |  | 1 |  |  | 2 | 1 |  |
|  | *Macariasignaria* |  |  | 1 |  |  |  |  |  |  |  |  |  |
|  | *Chiasmiaclathrata* | 1 | 4 | 2 |  | 7 | 1 | 2 | 17 | 2 | 1 |  | 1 |
|  | *Cepphisadvenaria* |  |  | 1 | 1 |  |  |  | 1 | 1 | 1 |  |  |
|  | *Plagodisdolablaria* |  |  | 1 |  |  |  |  |  |  |  | 1 |  |
|  | *Opisthograptisluteolata* |  |  |  |  |  |  |  |  |  |  |  |  |
|  | *Angeronaprunaria* |  |  | 8 | 1 |  |  | 1 |  | 4 | 2 | 5 | 3 |
|  | *Antonechlorissmaragdaria* |  |  |  |  |  |  |  |  |  |  |  | 1 |
|  | *Jodisputata* |  |  |  |  |  |  |  |  |  |  |  |  |
|  | *Hypomecisroboraria* |  |  |  |  |  |  |  | 1 | 4 | 3 | 8 | 3 |
|  | *Hypomecispunctinalis* |  |  | 2 |  |  |  |  |  | 1 | 1 | 4 | 2 |
|  | *Caberapussaria* |  | 1 |  |  |  | 1 |  |  | 2 |  | 1 |  |
|  | *Caberaexanthemata* |  |  |  |  |  |  |  | 1 | 1 | 2 |  |  |
|  | *Scopulaimmorata*^1^ |  | 1 |  |  | 1 |  | 1 | 2 | 2 | 3 | 1 |  |
|  | *Scopulaornata* |  |  |  |  |  |  |  |  |  |  |  |  |
|  | *Scopulaimmutata* |  |  | 5 |  |  |  | 1 |  |  |  |  |  |
|  | *Xanthorhoemontanata* |  |  | 2 | 8 |  |  |  | 1 | 1 |  |  |  |
|  | *Cosmorhoeocellata* |  |  |  |  |  |  |  |  |  |  |  |  |
|  | *Chloroclystatruncata* |  |  |  | 1 |  |  |  | 1 |  |  | 1 |  |
|  | *Eupitheciatripunctaria* |  |  |  |  |  |  |  |  | 1 |  |  |  |
|  | *Eupitheciateniuata* |  |  |  |  |  |  |  |  | 2 |  |  |  |
|  | *Eupitheciaplumbeolata* |  |  |  |  |  |  |  |  |  |  |  |  |
|  | *Hydraeliaflammeolaria* |  | 1 |  | 1 |  |  |  |  | 1 | 1 |  |  |
|  | *Hydraeliasylvata* |  |  |  |  |  |  |  |  |  |  |  |  |
|  | *Ectropiscrepuscualria* |  |  | 2 |  |  |  |  |  |  |  |  | 2 |
|  | *Timandracomae* |  |  | 1 |  | 4 |  |  |  |  |  |  |  |
|  | *Idea avarsata* |  |  | 1 |  |  |  |  |  | 1 | 2 |  | 1 |
|  | *Idea bisellata* |  |  |  |  |  |  |  |  |  |  | 2 |  |
|  | *Eulithisprunata* |  |  | 1 |  |  |  |  |  |  |  |  |  |
|  | *Eulithismellinata* |  |  |  |  |  |  |  |  | 1 | 1 |  |  |
|  | *Ecliptopteracapitata* |  |  | 5 | 3 |  |  |  | 1 |  |  |  | 1 |
|  | *Ligdiaadustata* |  |  | 2 |  |  |  |  |  | 1 |  |  |  |
|  | *Siona lineata* |  |  |  |  | 2 |  | 2 | 4 |  |  |  |  |
|  | *Perizomaalchemillata* |  |  |  |  |  |  |  |  |  |  | 1 | 1 |
|  | *Perizomafavofasciata* |  | 1 |  |  |  |  |  |  |  |  |  | 1 |
|  | *Plagodispulveraia* |  |  |  | 1 |  |  |  |  |  |  |  |  |
|  | *Mesolecaalbicillata* |  |  |  | 1 |  |  |  |  |  |  | 1 |  |
|  | *Rheumapteraundulata* |  |  |  | 1 |  |  |  |  |  |  |  |  |
|  | *Eustromareticulata* |  |  |  | 1 |  |  |  |  |  |  |  |  |
|  | *Alcisribeata* |  |  |  |  |  |  |  |  |  |  | 1 |  |
|  | *Alcisrependata* |  |  |  |  |  |  |  | 3 |  |  |  | 3 |
|  | *Camtogramabilineata* |  |  |  |  |  |  |  | 1 |  |  |  |  |
|  | *Aploceraplagiata*12.3***; **8.96** |  |  |  |  |  |  |  | 1 |  |  |  |  |
|  | *Pterapherapteryxsexalata* |  |  |  |  |  |  |  | 1 |  |  |  | 2 |
|  | *Petrophorachlorosata* |  |  |  |  |  |  |  |  | 23 | 12 |  |  |
|  | *Aethalurapunctulata* |  |  |  |  |  |  |  |  | 5 |  | 2 |  |
|  | *Cyclophoraalbipunctata* |  |  |  |  |  |  |  |  | 1 |  |  |  |
|  | *Cyclophoraquercimontaria* |  |  |  |  |  |  |  |  |  |  | 1 |  |
|  | *Cyclophorapendularia* |  |  |  |  |  |  |  |  |  |  |  | 1 |
|  | *Orthonamavitata* |  |  |  |  |  |  |  |  | 1 |  |  |  |
|  | *Catarhoecuculata* |  |  |  |  |  |  |  |  | 1 |  |  |  |
|  | *Catarrhoerubidata* |  |  |  |  |  |  |  |  |  |  |  | 1 |
|  | *Epirhoealternata* |  |  |  |  |  |  |  |  | 5 | 2 |  | 6 |
|  | *Epirrhoetartruensis* |  |  |  |  |  |  |  |  |  |  |  | 2 |
|  | *Hydriomenafurcata* |  |  |  |  |  |  |  |  | 1 |  | 1 | 1 |
|  | *Euchoecanebulata* |  |  |  |  |  |  |  |  | 1 |  |  | 6 |
|  | *Geometra papilionaria* |  |  |  |  |  |  |  |  |  |  | 1 |  |
|  | *Bupaluspiniaria* |  |  |  |  |  |  |  |  |  |  | 1 |  |
|  | *Comibenabajularia* |  |  |  |  |  |  |  |  |  |  | 1 |  |
|  | *Euphiaunangulata* |  |  |  |  |  |  |  |  |  |  | 2 |  |
|  | *Bistonbetularia* |  |  |  |  |  |  |  |  |  |  |  | 1 |
|  | *Colostygiapectinataria* |  |  |  |  |  |  |  |  |  |  |  | 1 |
|  | *Euphiaunangulata* |  |  |  |  |  |  |  |  |  |  |  | 1 |
|  | *Ashtenaalbulata* |  |  |  |  |  |  |  |  |  |  |  | 1 |
|  | *Macaraiaalternata* |  |  |  |  |  |  |  |  | 7 | 5 |  | 1 |
|  | *Chariospilatesexanthemata* |  |  |  |  |  |  |  |  |  | 2 |  |  |
| Lasiocampidae | *Macrothylaciarubi* |  |  |  |  |  |  |  |  |  | 1 |  |  |
|  | *Sphinx ligustri*^1^36-42*; **43.97** |  |  |  |  |  |  |  | 1 |  |  |  |  |
|  | *Eutrixpotatoria* |  |  |  |  |  |  |  |  | 1 | 1 |  |  |
|  | *Gastropachaqurecifolia* |  |  |  |  |  |  |  |  |  |  | 1 |  |
|  | *Lasiocampatrifolii* |  |  |  |  |  |  |  |  |  |  | 1 |  |
| Limantridae | *Caliterapudibunda* |  |  |  | 2 |  |  |  |  |  |  |  |  |
| Lomacodidae | *Apodalimacodes* |  |  | 1 |  |  |  |  |  |  |  | 31 |  |
| Noctuidae | *Lygephilapastinum* |  |  |  |  |  |  |  | 1 |  |  |  |  |
|  | *Lygephilaviciae* |  |  |  |  |  |  | 2 |  |  |  |  |  |
|  | *Autographa gamma*  15-16*/**; 16.2*** ^2^ |  |  | 1 |  |  |  |  |  |  |  |  |  |
|  | *Autographa jota* |  |  | 2 |  |  |  |  |  |  |  |  | 1 |
|  | *Abrostolatripartita*  12-13** |  |  | 2 |  |  |  |  |  |  |  |  |  |
|  | *Protodeltotepygarga* |  |  | 1 |  |  |  |  | 4 | 6 | 4 |  | 11 |
|  | *Pseudeustrotiacandidula* |  |  |  |  |  |  |  |  |  |  |  | 1 |
|  | *Cuculliaumbratica* ^2^ |  |  |  |  |  |  |  |  |  |  |  |  |
|  | *Hoplodrinablanda* |  |  |  |  |  |  |  |  |  |  |  |  |
|  | *Euplexialucipara* |  |  |  |  |  |  |  |  |  | 1 |  | 1 |
|  | *Apameacrenata* |  |  | 1 |  | 1 |  | 1 |  |  |  |  |  |
|  | *Apameamonoglypha*12.1*** |  |  |  |  | 4 |  | 4 |  |  |  |  |  |
|  | *Melanchrapisi* |  |  |  |  |  |  |  |  |  |  |  |  |
|  | *Polianebulosa* |  |  |  |  | 1 |  | 1 |  |  |  |  | 1 |
|  | *Oligiastrigilis* |  | 1 |  |  |  |  |  |  |  | 1 |  |  |
|  | *Oligiafasciuncula* |  |  |  |  |  |  |  | 2 | 1 |  |  |  |
|  | *Oligiaversicolor* |  |  |  |  |  |  |  |  |  |  |  | 1 |
|  | *Lacanobiathalassina* |  |  |  | 1 |  |  |  |  |  |  |  |  |
|  | *Lacanobiasuasa* |  |  |  |  |  |  |  |  |  |  |  |  |
|  | *Lacanobiasplendens* |  |  |  |  |  |  |  |  | 5 |  |  |  |
|  | *Discestratrifolii* |  |  |  |  |  |  |  | 1 |  |  | 3 |  |
|  | *Mythimnaconigera* |  |  | 1 |  | 6 |  | 2 |  |  |  |  |  |
|  | *Mythimnapallens* |  |  |  |  |  |  | 1 |  |  |  |  |  |
|  | *Mythimnaturca* |  |  | 1 |  | 2 |  | 1 | 1 |  |  |  | 4 |
|  | *Mythimnaimpura* |  |  |  |  |  |  |  |  |  |  |  |  |
|  | *Mythimnaalbipuncta* |  |  |  |  |  |  |  |  |  |  |  |  |
|  | *Mythimnapudorina* |  |  |  |  |  |  |  |  | 1 | 3 |  | 1 |
|  | *Mythimnaflammea* |  |  |  |  |  |  |  |  | 8 |  |  |  |
|  | *Mythimnaferrago* |  |  |  |  |  |  |  |  |  |  |  | 1 |
|  | *Axyliaputris* |  |  |  |  |  |  |  |  |  |  |  |  |
|  | *Ochropleuraplecta* |  |  |  |  |  |  |  |  |  |  |  |  |
|  | *Noctuapronuba*13-15**^2^ |  |  | 3 |  |  | 1 |  |  |  |  |  |  |
|  | *Diarsiabrunnea* |  |  |  |  |  |  |  |  |  |  |  |  |
|  | *Diarsiadahli* |  |  |  |  |  |  |  | 1 |  |  |  |  |
|  | *Diarsiamendica***7.27** |  |  | 3 | 18 | 1 | 1 | 2 |  |  |  |  |  |
|  | *Anaplectoidesprasina* |  |  |  |  |  |  |  |  |  |  |  |  |
|  | *Rhyaciasimulans* |  |  |  |  |  |  |  |  |  |  |  |  |
|  | *Agrotisexclamationis* |  |  | 22 |  | 11 |  | 5 |  | 1 | 1 |  |  |
|  | *Acronictarumicis* |  |  | 1 |  |  |  |  |  |  |  |  |  |
|  | *Acronictaleporina* |  |  |  |  |  |  |  |  | 1 |  |  |  |
|  | *Acronictacuspis* |  |  |  |  |  |  |  |  | 1 | 1 |  |  |
|  | *Acronictamegacephala* |  | 1 |  |  | , |  | 1 |  | 1 | 2 | 4 | 10 |
|  | *Herminia grisealis* |  |  | 3 |  |  |  |  | 1 |  |  |  |  |
|  | *Herminia trasiclinnaris* |  |  |  |  |  |  |  |  | 6 | 5 | 5 | 4 |
|  | *Ligephilaviciae* |  |  | 1 |  |  |  |  |  |  |  |  |  |
|  | *Diachrysiachrysitis*  15-16** |  |  | 1 |  |  |  |  |  |  | 1 |  |  |
|  | *Cryphiaalgae* |  |  | 2 |  |  |  |  |  |  |  |  |  |
|  | *Charanycatrigrammica* |  |  | 5 |  |  |  |  |  |  |  |  |  |
|  | *Dypterygiascabriuscula* |  |  | 3 |  |  |  |  | 2 |  |  |  |  |
|  | *Rusina ferruginea* |  |  | 3 |  | 3 |  | 4 | 2 |  |  | 1 | 1 |
|  | *Hada plebeja***9.66** |  |  | 3 | 1 |  |  | 1 | 1 |  |  |  |  |
|  | *Xestiatriangulum* |  |  | 2 |  |  |  | 6 | 3 |  |  | 3 | 7 |
|  | *Xestia baj* |  |  |  |  |  |  |  |  |  |  |  | 1 |
|  | *Rivulasericealis*5** |  |  |  |  |  |  |  | 1 | 1 | 1 |  |  |
|  | *Polypogontentacularia* |  |  |  |  |  |  |  | 1 |  |  | 3 |  |
|  | *Bena bicolorana* |  |  |  |  |  |  |  | 1 |  |  |  |  |
|  | *Deltotebankiana* |  |  |  |  |  |  |  |  | 4 | 1 |  | 2 |
|  | *Deltoteuncula* |  |  |  |  |  |  |  |  |  |  |  | 1 |
|  | *Elaphriavenustula* |  |  |  |  |  |  |  |  | 1 | 1 |  | 1 |
|  | *Paradiarsiapunicea* |  |  |  |  |  |  |  |  | 1 |  |  |  |
|  | *Hadenabicruris* |  |  |  |  |  |  |  |  |  |  | 1 |  |
|  | *Hadenarivularis***36.7** |  |  |  |  |  |  |  |  |  |  |  | 1 |
|  | *Zonclognathatarsipennalis* |  |  |  |  |  |  |  |  |  |  | 2 |  |
|  | *Rivilasericealis* |  |  |  |  |  |  |  |  |  |  |  | 1 |
|  | *Hypenacrasalis* |  |  |  |  |  |  |  |  |  |  |  | 1 |
|  | *Hypenaproboscidalis* |  |  |  |  |  |  |  |  |  |  |  | 1 |
|  | *Ygogasignifera* |  |  |  |  |  |  |  |  |  |  |  | 1 |
|  | *Colobochylasalicalis* |  |  |  |  |  |  |  |  |  |  |  | 1 |
|  | *Macrochilocribrumalis* |  |  |  |  |  |  |  |  |  |  |  | 1 |
|  | *Thetellafluctuosa* |  |  |  |  |  |  |  |  |  |  |  | 1 |
|  | *Tracheaatriplicis* |  |  |  |  |  |  |  |  |  | 1 |  |  |
| Nolidae | *Eariaschlorana* |  |  |  |  |  |  |  |  |  |  |  |  |
| Notodontidae | *Gluphisacrenata* |  |  |  |  |  |  |  |  |  |  | 9 | 6 |
|  | *Stauropus fagi* |  |  | 1 |  |  |  |  |  | 1 | 3 | 2 |  |
|  | *Phalerabucephala* |  |  | 1 |  | 5 |  | 2 |  |  |  | 3 | 3 |
|  | *Ptilodoncapucina* |  |  |  | 1 |  |  |  |  |  |  |  |  |
|  | *Drymoniadodonea* |  |  |  |  |  |  |  |  |  |  | 3 |  |
|  | *Closterapigra* |  |  |  |  |  |  |  |  | 1 |  |  |  |
| Sphingidae | *Smerinthusocellata* |  |  | 1 |  |  |  |  |  |  |  |  | 4 |
|  | *Lathoepopuli* |  |  | 3 |  |  |  |  |  |  | 3 |  |  |
|  | *Hyloicuspinastri*^1^*;* 29.5***; **28.24; 28.07** |  |  |  |  |  | 1 |  | 1 | 1 |  | 3 |  |
|  | *Deilephilaelpenor*  250-280*; 22.5***;**23.7** |  |  |  |  |  |  |  |  | 1 |  |  |  |
|  | | | | | | | | | | | | | |
| The number of species | ∑ 170 | 2 | 8 | 45 | 17 | 19 | 6 | 25 | 34 | 49 | 35 | 42 | 56 |
| The number of individuals | ∑ 920 | 2 | 15 | 119 | 53 | 61 | 6 | 56 | 84 | 142 | 77 | 147 | 158 |

Insect species observed on *Platantherabifolia*/*chlorantha* inflorescences by^1^Boberg et al. 2014; ^2^ Esposito et al. 2017; tongue length (mm)*Willmer 2011,

** Sexton 2014, ***Nilsson 1983; in bold: our observations
